# Bacterial inoculation drives microbiome-mediated resistance to a soil-borne pathogen in wheat

**DOI:** 10.1038/s41522-026-01021-8

**Published:** 2026-05-26

**Authors:** Caroline Sayuri Nishisaka, Hélio Danilo Quevedo, Thierry Alexandre Pellegrinetti, Fernanda de Almeida Godoy, Maike Rossmann, Lucas William Mendes, Rodrigo Mendes

**Affiliations:** 1 Embrapa Environment, Jaguariúna, SP Brazil; 2 Center for Nuclear Energy in Agriculture, University of São Paulo, Piracicaba, SP Brazil; 3 College of Agriculture “Luiz de Queiroz”, University of São Paulo, Piracicaba, SP Brazil

**Keywords:** Microbiology, Plant sciences

## Abstract

Soil microbiomes are fundamental to plant health, mediating nutrient cycling, stress tolerance, and pathogen defense. However, soil-borne pathogens such as *Bipolaris sorokiniana* severely constrain wheat productivity. Despite growing interest, the mechanisms by which beneficial bacterial inoculation reshapes rhizosphere microbial communities to enhance disease resistance remain poorly understood. Here, we isolated three bacterial strains, *Streptomyces virginiae* CMAA1738, *Paenibacillus ottowii* CMAA1739, and *Pseudomonas inefficax* CMAA1741, with antagonistic activity against *B. sorokiniana*, and evaluated their effects on wheat under controlled conditions. Through plant bioassays, bacterial inoculation reduced disease severity by ~60% and promoted root growth. Metataxonomic and metagenomic analyses revealed shifts in the structure and functional potential of the rhizosphere microbiome. Structural equation modeling indicated that inoculation was the primary driver of microbiome restructuring and disease suppression. Notably, inoculation restored the diversity of plant growth-promoting genes and biosynthetic gene clusters reduced by pathogen infection, enriching functions associated with stress tolerance, nutrient metabolism, and secondary metabolite production. In addition, Random Forest analysis revealed that variation in disease severity under pathogen pressure was associated with differences in bacterial community composition. Together, these findings demonstrate that bacterial inoculation can restructure the rhizosphere microbiome and restore key functional traits linked to plant resilience.

## Introduction

Soil is one of the most complex ecosystems on Earth, characterized by rich microbial diversity and a great reservoir of genetic and metabolic potential^1,2^. Within this highly diverse community, countless ecological processes co-occur, supporting multiple ecosystem functions and maintaining soil multifunctionality^3–5^. A subset of the soil microbiome plays particularly crucial roles in plant growth and protection by contributing to nutrient cycling, defense against pathogens and pests, and mitigation of abiotic stresses^6,7^. These interactions are especially intense in the rhizosphere, a dynamic hotspot of microbial activity and diversity enriched by plant exudates and inhabited by bacteria, fungi, and other microbial groups^7,8^.

In this highly interactive environment, the success of invasion, whether by pathogens or beneficial microbes, is determined by the diversity, composition, and competitive structure of the resident microbial community^9–11^. According to the diversity-invasibility framework^12^, highly diverse microbiomes tend to resist colonization through niche pre-emption and intense resource competition, whereas simplified communities may leave ecological space available for invasion^13,14^. These dynamics can influence plant health in contrasting ways: while diverse communities may suppress pathogen establishment, they can also hinder the colonization of beneficial inoculants. Nishisaka et al.^15^ demonstrated that both *Bipolaris sorokiniana* and the biocontrol agent *Pseudomonas inefficax* CMAA1741 are affected by soil microbial diversity, with pathogen suppression in low-diversity soils largely attributed to enhanced establishment of the inoculant. These findings suggest that biocontrol efficacy depends on the balance between the resident microbiome’s resistance to invasion and the inoculant’s capacity to exploit available ecological niches.

Wheat (*Triticum aestivum*) was selected as the model host due to its global relevance and susceptibility to *Bipolaris sorokiniana* (teleomorph *Cochliobolus sativus*), a soil-/seed-borne hemibiotroph that causes spot blotch, common root/crown rot, seedling blight, and black point, with substantial yield penalties, particularly in warm or water-limited environments^16,17^. Heavy reliance on fungicides and other chemical inputs carries ecological costs-non-target effects, such as soil^18^ and water contamination^19^, and selection for resistance^20^, rendering these approaches environmentally unsustainable. In this context, beneficial rhizobacteria (e.g., *Pseudomonas* sp.) offer microbiome-informed alternatives that can reshape rhizosphere communities and constrain pathogen impact through complementary, community- and host-mediated processes^15,21,22^. Thus, these traits classify microbial inoculants as effective disease-management options that mitigate environmental contamination relative to conventional chemical controls^23,24^.

Despite increasing recognition of the rhizosphere microbiome’s role in plant health, the mechanisms by which beneficial bacteria modulate microbial community structure and function to suppress pathogens remain poorly resolved. Understanding these interactions is critical for developing inoculation strategies that promote microbiome-mediated disease resistance in crops such as wheat. In this study, we investigated how antagonistic bacterial inoculation influences the rhizosphere microbiome and the functional mechanisms underlying plant protection. Following an initial screening of multiple bacterial isolates for antagonistic activity, we selected three strains, *Streptomyces virginiae* CMAA1738, *Paenibacillus ottowii* CMAA1739, and *Pseudomonas inefficax* CMAA1741, for detailed analysis. We combined metataxonomic and metagenomic approaches to assess shifts in rhizosphere community composition and to characterize changes in the abundance and diversity of plant growth-promoting (PGP) genes within the resident microbiome. By integrating metataxonomic and metagenomic approaches, we assessed shifts in rhizosphere community composition and functional profiles, focusing on plant growth-promoting and biosynthetic traits. This combined analysis allowed us to elucidate how beneficial bacteria restructure microbial communities and restore key functions associated with disease suppression.

## Results

### In vitro screening of isolates and their antagonistic activity against Bipolaris sorokiniana

The screening results of 30 isolates for beneficial traits through biochemical tests are listed in Table S1. Strains exhibiting Indole-3-acetic acid (IAA) production, phosphate solubilization, and antagonism against *B. sorokiniana* were considered candidates for further experiments. Among them, five strains tested positive for these traits, and three were selected for additional testing based on their superior IAA production and phosphate solubilization. The selected isolates, *Streptomyces virginiae* strain CMAA1738, *Paenibacillus ottowii* strain CMAA1739, and *Pseudomonas inefficax* strain CMAA1741 (hereafter referred to as *Sv*, *Po*, and *Pi*, respectively), also underwent whole-genome sequencing for improved taxonomic assignment^25^.

**Fig. 1:**
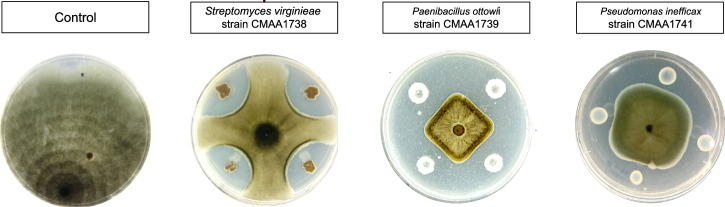
In vitro antagonism test of three bacterial strains, *Streptomyces virginiae* strain CMAA1738 (*Sv*), *Paenibacillus ottowii* strain CMAA1739 (*Po*), and *Pseudomonas inefficax* strain CMAA1741 (*Pi*), against *Bipolaris sorokiniana*, along with a control (*B. sorokiniana* alone), observed at 7 days. The inhibition zones around the bacterial colonies highlight their antagonistic potential against the pathogen.

The production of 1.15, 1.24, and 4.6 µg ml^–1^ of IAA was observed in *Sv*, *Po*, and *Pi*, respectively. Phosphate solubilization also showed the highest concentration in *Pi* (0.52 µg ml^–1^), followed by concentrations of 0.37 µg ml^–1^ for both *Sv* and *Po*. All three strains demonstrated effective inhibition of *B. sorokiniana* growth (Fig. 1).

Additionally, the nitrogen fixation test was conducted, and, among the three bacterial strains in this study, only the *Sv* did not exhibit nitrogen fixation activity (Table S1).

Thus, based on these traits, particularly their antagonistic activity against *B. sorokiniana*, these three bacterial strains were selected for the *in planta* bioassay, focusing on disease suppression.

### Disease suppression and beneficial potential of bacterial isolates

**Fig. 2:**
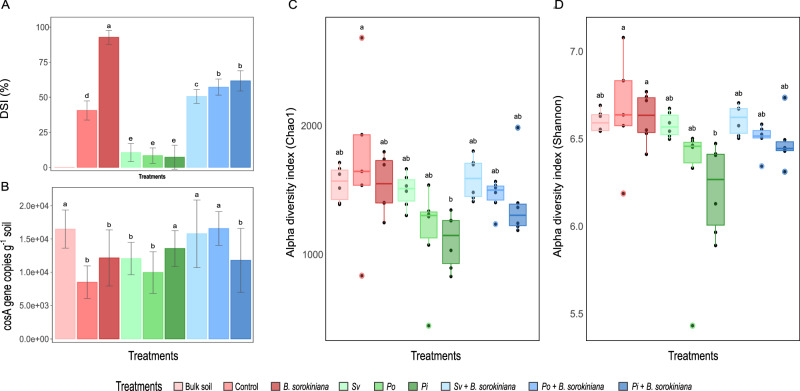
Plant bioassay, pathogen detection, and bacterial alpha diversity. **A** Disease severity index (DSI) of wheat plants treated with beneficial bacteria, inoculated or not with the pathogen *Bipolaris sorokiniana*, and control treatments. DSI values ranging from 0 to 100%. **B** *Bipolaris sorokiniana cos*A gene quantification by Real-Time Polymerase Chain Reaction (qPCR). The Scott-Knott statistical test (*p* < 0.05) was performed to compare the means, and different uppercase letters indicate significant differences. **C** Alpha diversity of bacterial community based on Chao1 index in different treatments. **D** Alpha diversity of bacterial community based on Shannon index in different treatments. HDS test applied considering significant differences with *p* < 0.05. Bulk soil (soil without plant influence), control (non-treated plants), *Bipolaris sorokiniana* (plants inoculated with the pathogen), *Sv* (plants inoculated with *Streptomyces virginiae* strain CMAA1738), *Po* (plants inoculated with *Paenibacillus ottowii* strain CMAA1739), *Pi* (plants inoculated with the *Pseudomonas inefficax* strain CMAA1741), *Sv* + *B. sorokiniana* (plants inoculated with the S. virginiae and with *B. sorokiniana*), *Po* + *B. sorokiniana* (plants inoculated with the *P. ottowii* and with *B. sorokiniana*), and *Pi* + *B. sorokiniana* (plants inoculated with the *P. inefficax* and with *B. sorokiniana*).

Infection with the pathogen *B. sorokiniana* significantly (*p* < 0.05) increased disease incidence in wheat compared to other treatments (Fig. 2A). However, plants from seeds treated with beneficial bacteria showed a reduction of ~60% in disease severity index (DSI) when compared to plants inoculated with *B. sorokiniana* alone (Fig. 2A). For pathogen quantification, bulk soil was included as an additional treatment, showing a high number of *cos*A gene, indicating a natural presence of resident *B. sorokiniana* in the soil (Fig. 2B). When compared with bulk soil, plant cultivation reduced *cos*A gene copy numbers in the control treatments (Fig. 2B). In contrast, *cos*A abundance varied among bacterial treatments, with three treatments (*Pi*, *Sv* + *B. sorokiniana*, and *Po* + *B. sorokiniana*) showing significantly higher gene copy numbers relative to *B. sorokiniana* alone. Despite this increase, disease severity was reduced in inoculated plants. No significant correlation was observed between *cos*A gene copy number and DSI (Spearman’s ρ = 0.09, *p* = 0.556), indicating that variation in this functional gene alone does not explain disease outcomes across treatments.

Plant shoots dry mass showed no significant differences among treatments (Fig. S1A), whereas root dry mass significantly increased under *Pi* inoculation (Fig. S1B). Conversely, plant height increased in all bacterial-inoculated treatments, with the greatest heights observed under *Pi* alone and *Po* + *B. sorokiniana* treatments (Fig. S2A). In addition, chlorophyll content significantly decreased in all treatments compared to the control.

### Impact of inoculation and infection on microbiome assembly

Bacterial alpha diversity showed limited variation among treatments. Most treatments displayed values statistically similar to the control (groups “a” or “ab”), whereas only the *Pi* treatment differed significantly for the Chao1 and Shannon indices (Fig. 2C). The most pronounced reduction was observed under *Pi* treatment, while *B. sorokiniana* had the least impact, as indicated by both Chao1 and Shannon indices. In contrast, fungal alpha diversity remained statistically unchanged across treatments (Fig. S3).

**Fig. 3:**
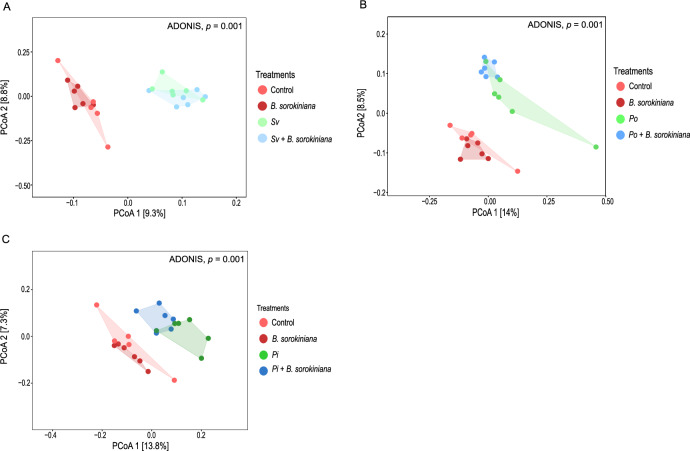
Principal coordinate analysis (PCoA) of rhizosphere bacterial communities under different treatments. **A** PCoA of rhizosphere bacterial communities in plants treated with *S. virginiae* strain CMAA1738, with or without *Bipolaris sorokiniana* infection. **B** PCoA of rhizosphere bacterial communities in plants treated with *P. ottowii* strain CMAA1739, with or without *B. sorokiniana* infection. **C** PCoA of rhizosphere bacterial communities in plants treated with *P. inefficax* strain CMAA1741, with or without *B. sorokiniana* infection. PCoAs were based on 16S rRNA gene amplicons, and statistical significance was assessed using Adonis (*p* < 0.05, 999 permutations; see Table S2). Control = water-treated plants; *Sv* = *S. virginiae* strain CMAA1738; *Po* = *P. ottowii* strain CMAA1739; *Pi* = *P. inefficax* strain CMAA1741; *B. sorokiniana* = plants infected with *B. sorokiniana*; *Sv* or *Po* or *Pi* + *B. sorokiniana* = plants inoculated with bacterial strain and pathogen.

Bray-Curtis-based PCoA revealed treatment-dependent clustering of bacterial communities (Fig. 3), indicating shifts in overall rhizosphere community composition when beneficial bacteria were inoculated. Inoculation with *Sv*, *Po*, and *Pi* each resulted in microbial communities that were distinct from the control (Fig. 3; Table S2). *Po* produced the largest shift in alpha diversity, which was significantly different from the control (Fig. 2C). Likewise, this treatment also differed significantly in beta diversity relative to the other treatments (Fig. 3E; Table S2).

Among the disease-suppressive treatments (bacterial inoculant + pathogen), *Pi* + *B. sorokiniana* was the only one that significantly altered the community compared to *Pi* alone (Fig. 3E). Both *Sv* (Fig. 3A) and Po (Fig. 3C), also suppressive treatments, assembled communities distinct from the control and *B. sorokiniana* alone (Table S2). A similar pattern was observed for the *Pi* + *B. sorokiniana* treatment, which also differed from these same treatments (Table S2).

**Fig. 4:**
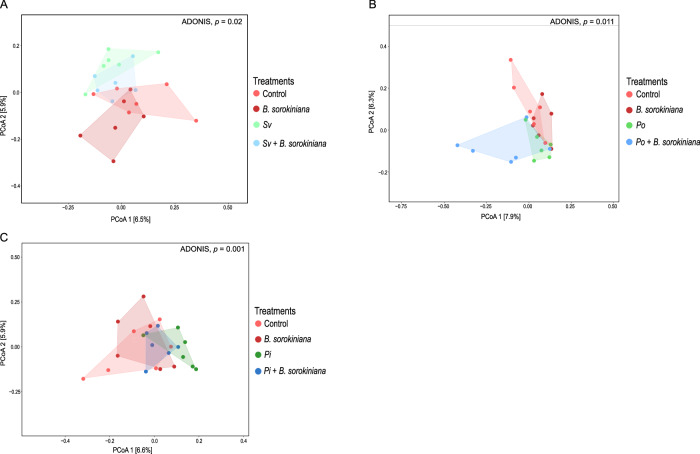
Principal coordinate analysis (PCoA) of rhizosphere fungal communities under different treatments. **A** PCoA of rhizosphere fungal communities in plants treated with *Sv*, with or without *Bipolaris sorokiniana* infection. **B** PCoA of rhizosphere fungal communities in plants treated with *Po*, with or without *B. sorokiniana* infection. **C** PCoA of rhizosphere fungal communities in plants treated with *Pi*, with or without *B. sorokiniana* infection. PCoAs were based on ITS region amplicons, and statistical significance was assessed using Adonis (*p* < 0.05, 999 permutations; see Table S2). Control = water-treated plants; *Sv* = *S. virginiae* strain CMAA1738; *Po* = *P. ottowii* strain CMAA1739; *Pi* = *P. inefficax* strain CMAA1741; *B. sorokiniana* = plants infected with *B. sorokiniana*; *Sv* or *Po* or *Pi* + *B. sorokiniana* = plants inoculated with bacterial strain and pathogen.

Fungal communities were overall less affected by the treatments compared to bacteria. No significant differences were detected in alpha diversity (Chao1 and Shannon indices; Fig. S3), suggesting a higher resilience or lower responsiveness of fungal assemblages to the inoculations. Nonetheless, beta-diversity analysis revealed pairwise differences (Fig. 4; Table S3). In particular, *Po* stood out as the treatment with the greatest number of significant pairwise contrasts, differing from nearly all other treatments except *B. sorokiniana* versus the control. Additional significant pairwise differences were detected; for instance, fungal communities under *B. sorokiniana* alone were distinct from those in the *Po* + *B. sorokiniana* treatment (*p* = 0.008) (Fig. 4B).

### Structural equation modeling (SEM)

**Fig. 5:**
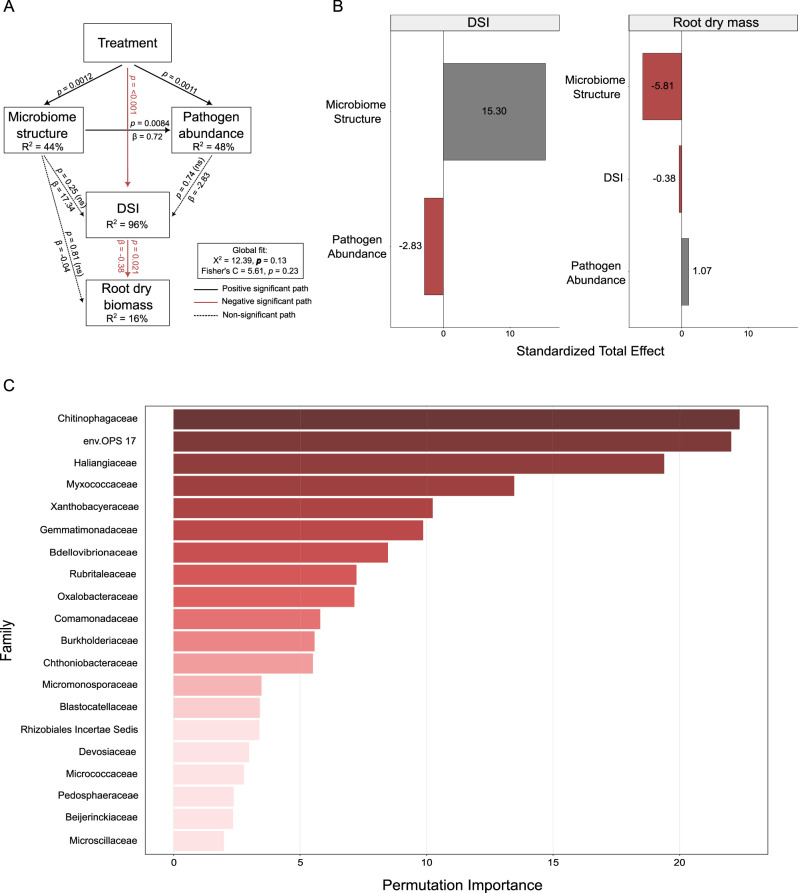
Integrative analysis of disease suppression using structural equation modeling (SEM) and Random Forest. **A** Conceptual workflow and results of the SEM describing the relationships between treatment (control, pathogen infected-, bacterial inoculated-, and bacteria + pathogen-based treatments) rhizosphere microbiome structure (PCoA1), pathogen abundance (*Bipolaris sorokiniana cos*A gene quantified by qPCR), disease severity index (DSI), and root dry mass. Solid arrows indicate significant paths (*p* < 0.05), whereas dashed arrows represent non-significant relationships. Numbers adjacent to arrows indicate standardized path coefficients (β) and associated *p*-values. Coefficients of determination (R^2^) are shown within response variables. β values are shown only for continuous predictors; treatment effects are presented as overall significance due to their categorical nature. **B** Standardized total effects of microbiome structure, pathogen abundance, and disease severity index on plant performance variables, illustrating their relative contributions to variation in DSI and root dry mass. **C** Random Forest regression identifying bacterial families associated with variation in disease severity among pathogen-infected treatments (*B. sorokiniana*, *Sv* + *B. sorokiniana*, *Po* + *B. sorokiniana*, and *Pi* + *B. sorokiniana*). Bars represent permutation importance scores of the top bacterial families contributing to disease severity prediction. The model explained 48% of the variation in disease severity (out-of-bag R^2^ = 48%).

Structural equation modeling (SEM) was used to integrate microbiome structure (PCoA1), pathogen abundance (qPCR of *cos*A gene), disease severity index (DSI), and plant performance (root dry mass) into a causal framework (Fig. 5A). The model showed a good fit to the data (X^2^ = 12.39, *p* = 0.135; Fisher’s C = 5.61, *p* = 0.23), showing that the hypothesized relationships adequately explained the observed patterns. Inoculation treatment significantly influenced microbiome structure (PCoA1; *p* = 0.0012; R^2^ = 44%) and pathogen abundance (*p* = 0.0011), demonstrating that the introduced strains strongly reshaped rhizosphere microbial communities and pathogen dynamics.

Microbiome structure had a significant positive effect on pathogen abundance (β = 0.72, *p* = 0.008), suggesting that shifts in bacterial community composition were associated with changes in pathogen proliferation. Furthermore, disease severity was mainly driven by the inoculation treatment (*p* < 0.001), while the direct effects of pathogen abundance and microbiome structure were not statistically significant. The model explained a large proportion of the variation in disease severity (R^2^ = 96%). Root dry mass was negatively associated with disease severity (β = –0.38, *p* = 0.021), although the model explained only a modest proportion of the variation in root biomass (R^2^ = 16%). Analysis of total standardized effects showed that microbiome structure influenced disease severity both directly and indirectly through pathogen abundance, while disease severity exerted the strongest total effect on root biomass (Fig. 5B).

### Bacterial taxa associated with disease suppression under pathogen pressure

Random Forest regression was applied to identify bacterial taxa associated with variation in disease severity among pathogen-inoculated treatments (*B. sorokiniana*, *Sv* + *B. sorokiniana*, *Po* + *B. sorokiniana*, and *Pi* + *B. sorokiniana*). The model showed potential predictive performance, explaining 48% of the observed variation in disease severity (out-of-bag R² = 0.48). Permutation tests confirmed that this predictive power was not attributable to random structure in the dataset (permuted model R^2^ = –0.03). Variable importance analysis based on permutation importance scores identified several bacterial families contributing strongly to disease severity prediction (Fig. 5C).

Among these, Chitinophagaceae showed the highest importance value, followed by env.OPS 17 (member of Sphingobacteriales order), Haliangiaceae, Myxococcaceae, and Xanthobacteraceae. Additional families with notable contributions included Gemmatimonadaceae, Bdellovibrionaceae, Rubritaleaceae, Oxalobacteraceae, Comamonadaceae, and Burkholderiaceae.

Although importance values varied among taxa, most of predictive features were concentrated within a limited subset of bacterial families, which can indicate that disease severity variation among pathogen-inoculated treatments was associated with specific components of the rhizosphere bacterial community. The distribution of permutation importance scores across the top bacterial families is shown in Fig. 5C.

### Genetic characterization of the beneficial bacterial strains

**Fig. 6:**
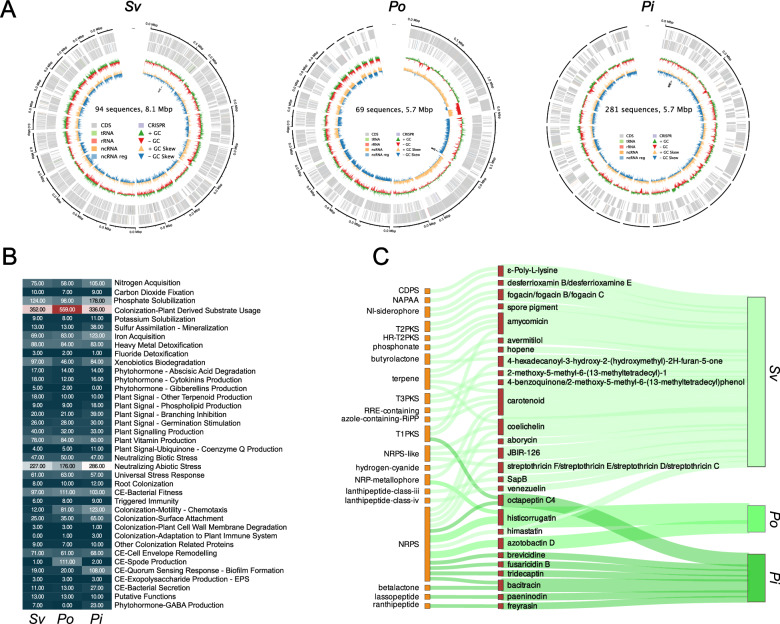
Genome characteristics, plant-beneficial traits, and biosynthetic potential of bacterial isolates. **A** Circular genome maps of isolates *Sv*, *Po*, and *Pi* generated using the Genovi pipeline. Genomic features including coding sequences (CDS), tRNAs, rRNAs, GC content, GC skew, CRISPR loci, and non-coding RNA regions are indicated. *Sv* displayed the largest genome (8.1 Mbp), followed by *Po* (5.7 Mbp) and *Pi* (5.7 Mbp), with differing contig numbers and structural organization. **B** Heatmap of plant growth-promoting (PGP) functional categories predicted by PGPg_finder against the PLaBAse database. Functional traits include nutrient acquisition, phytohormone production, stress responses, plant signaling modulation, biofilm formation, and microbial fitness. Values represent the number of annotated genes within each functional category per genome. **C** Biosynthetic gene clusters (BGCs) identified by antiSMASH and visualized with BiG-SCAPE.

The genome assemblies previously deposited by Nishisaka et al.^25^ were reannotated to enable a more detailed functional exploration of BGCs and PGP traits. The circular genomic maps (Fig. 6A) illustrate the chromosomal organization and distribution of major functional categories. BGC profiling revealed distinct secondary metabolite potentials among isolates (Fig. 6B). *Sv* encoded the most diverse set of clusters, including NRPS, PKS, CDPS, terpene, and RiPP classes, with predicted products such as avermirol, hopene, coelichelin, and other antimicrobial compounds. *Po* and *Pi* harbored fewer but ecologically relevant clusters, including himastatin, azotobactin, and freyrasin, indicating complementary functional roles within the soil environment.

Also, the genomes exhibited a wide range of PGP-related genes (Fig. 6C). *Pi* encoded the highest number of PGP-associated traits, including nitrogen acquisition, phosphate solubilization, sulfur assimilation, iron uptake, abiotic stress tolerance, chemotaxis, biofilm formation, secretion systems, and GABA production. *Po* was enriched in genes related to plant-derived substrate usage, vitamin biosynthesis, biotic stress tolerance, and sporulation, whereas *Sv* contained more genes linked to heavy metal detoxification, xenobiotic degradation, and phytohormone synthesis.

### Impact of beneficial bacterial inoculation on microbiome plant growth-promoting genes and biosynthetic gene clusters

**Fig. 7:**
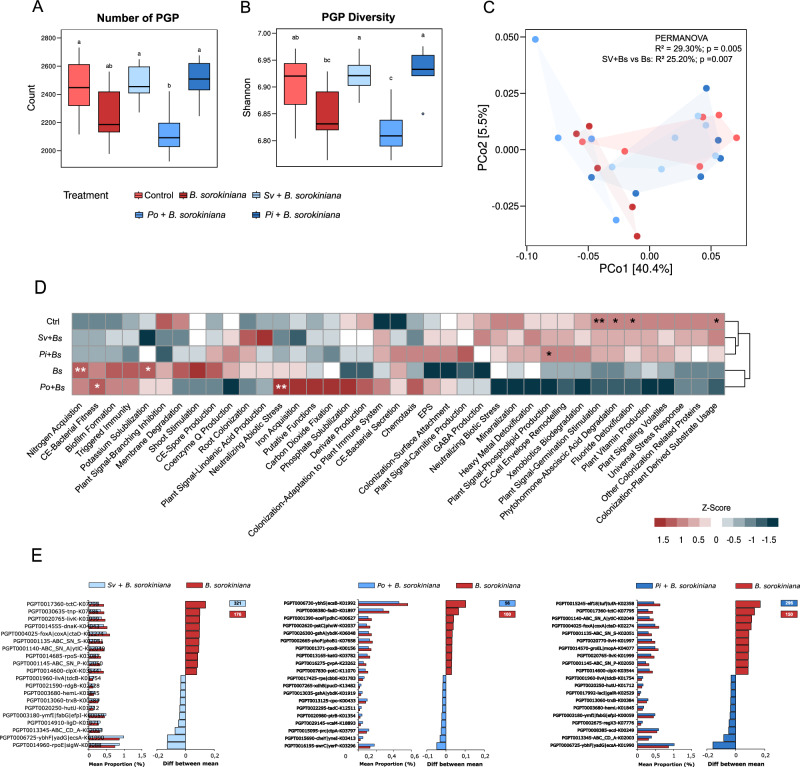
Plant growth-promoting (PGP) genes and functional shifts in root-associated microbial communities following bacterial inoculation. **A** Total number of predicted PGP genes and **B** Shannon diversity index across treatments. Different letters indicate statistically significant differences (Tukey’s HSD, *p* < 0.05). **C** Principal coordinate analysis (PCoA) of Bray-Curtis dissimilarities based on PGP gene profiles. **D** Heatmap of Level 3 of PLaBAse categories, showing treatment-specific shifts in gene abundance (Z-scores). Asterisks indicate significant differences (Kruskal-Wallis test, *p* < 0.05). **E** Differential abundance of Level 5 PGP genes (paired comparison) between each bacterium + pathogen treatment and *B. sorokiniana* (*Bs*).

For shotgun metagenomic sequencing, we selected the treatments most relevant to disease suppressiveness: Control, *B. sorokiniana*, *Sv* + *B. sorokiniana*, *Po* + *B. sorokiniana*, and *Pi* + *B. sorokiniana*. These treatments were chosen to specifically evaluate how pathogen infection + inoculation with antagonistic strains influence rhizosphere functional potential. The inoculation with bacterial isolates significantly affected the distribution and functional profile of PGP genes and BGCs within the whole rhizosphere microbial communities. PGP gene profiles showed changes in both richness and diversity (Fig. 7A, B). Infection with *B. sorokiniana* did not result in statistically significant differences in the number or diversity of PGP genes compared to the control in pairwise comparisons. However, the Po + *B. sorokiniana* treatment showed significantly lower PGP gene counts and Shannon diversity relative to the control. Treatments with isolates *Sv* and *Pi* mitigated this reduction, restoring gene counts and Shannon PGP diversity to levels comparable to the control (Fig. 7A, B). Beta diversity analysis based on Bray-Curtis distances revealed distinct clustering of treatments, particularly between *B. sorokiniana* alone and *Sv*-treated plants (Fig. 7C), as confirmed by PERMANOVA (*p* = 0.007).

At the functional level (Fig. 7D), the heatmap of Lv3 PGP categories revealed several functions that were significantly altered (indicated by asterisks) across treatments. In a more detailed view, plants treated with beneficial microbes and inoculated with the pathogen showed an enrichment of specific PGP-related genes when compared to *B. sorokiniana*-only treatment (Fig. 7E). For *Sv* + *B. sorokiniana*, the most enriched functions included genes associated with amino acid biosynthesis (*ilvA*), osmoprotectant synthesis (*hutU*, *hemL*, *trxB*), and stress tolerance (*rdgB*), in addition to genes involved in ABC transporters (*ABC_CD_A*, *ybhF/yadG/ecsA*) and transcriptional regulation (*rpoE/sigW*), which are known to contribute to membrane stabilization and stress responses. In the *Po* + *B. sorokiniana* treatment, genes involved in redox balance (*rpe, gshA*), transcriptional regulation (*vcaM, cpo*), and DNA transfer systems (*tadC, ptrB*) were more abundant. Genes associated with protein turnover (*prc*) and chemotaxis (*cheY*, *swrC*) were also enriched. In the *Pi* + *B. sorokiniana* treatment, key enriched genes included those involved in amino acid metabolism (*ilvA*), osmoprotectant and antioxidant production (*hutU*, *trxB*, *hemL*), and regulatory functions (*lacI*, *regX3*, *acd*), along with membrane transport (*ABC_CD_A*, *ybhF/yadG/ecsA*).

**Fig. 8:**
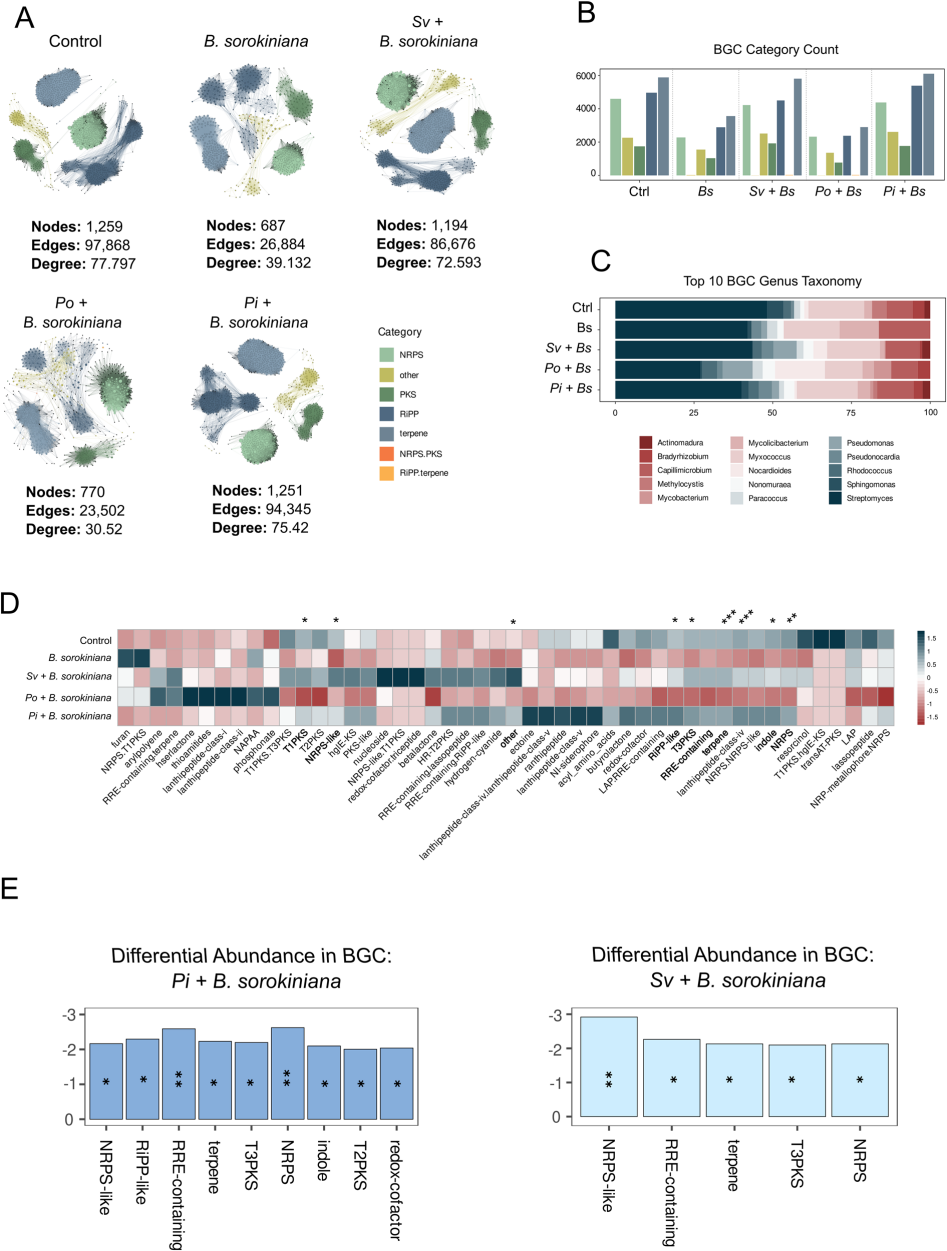
Biosynthetic gene clusters (BGCs) structure, diversity, and differential abundance across microbial communities. **A** BiG-SCAPE network analysis of BGCs across treatments. Each node represents a BGC, colored by biosynthetic class. **B** Total count of BGC categories across treatments. **C** Barplot showing the relative abundance of the top 10 BGC-producing genera. **D** Z-score heatmap of BGC classes (antiSMASH output), highlighting treatment-specific differences. Asterisks indicate significant variation compared to *B. sorokiniana* (Kruskal–Wallis test, *p* < 0.05). **E** Pairwise comparison of BGC class abundance in the *Pi* + *B. sorokiniana* and *Sv* + *B. sorokiniana* treatments, showing significantly increased abundance of NRPS-like, RiPP, terpene, and polyketide clusters relative to *B. sorokiniana* alone.

The BGC analysis revealed clear differences in community structure, biosynthetic capacity, and taxonomic composition across treatments. Network analysis (Fig. 8A) showed that inoculation with *B. sorokiniana* reduced BGC network size, connectivity, and complexity, as evidenced by decreases in node count, edge number, and degree centrality. In contrast, pathogen infection following beneficial bacterial inoculation, particularly *Sv* and *Pi*, restored these network properties, especially within key biosynthetic classes such as NRPS, PKS, RiPP, and terpene clusters.

Quantitative comparisons of BGC category counts (Fig. 8B) confirmed these observations, with the antagonist + pathogen treatments showing increased abundance of NRPS, RiPP, and terpene clusters compared to the *B. sorokiniana*-only treatment. Taxonomic profiling of BGC-producing genera (Fig. 8C) showed that *Streptomyces*, *Myxococcus*, and *Capillimicrobium* were dominant across all treatments. Specific taxa were enriched in particular treatments: *Mycolicibacterium* was more abundant in the *B. sorokiniana* treatment, while *Pseudomonas* and *Nocardioides* were enriched in *Sv* and *Po* treatments, respectively.

The heatmap of BGC categories (Fig. 8D) revealed significant shifts in abundance across treatments. Inoculation with bacterial isolates + infection with pathogen increased the coverage of several BGC classes, including T1PKS, NRPS-like, RiPP-like, T3PKS, terpene, indole, and NRPS clusters. Pairwise comparisons (Fig. 8E) confirmed that these enrichments were statistically significant when compared to *B. sorokiniana* alone. The *Pi* + *B. sorokiniana* treatment showed the most pronounced increases, with significant enrichment in NRPS-like, RiPP-like, terpene, T3PKS, NRPS, indole, T2PKS, and RRE-containing clusters. Similarly, *Sv* + *B. sorokiniana* significantly increased NRPS-like, RRE-containing, terpene, T3PKS, and NRPS clusters, suggesting that both isolates enhanced the biosynthetic potential of the microbial community.

## Discussion

The three bacterial isolates evaluated in this study belong to genera that are widely recognized for their beneficial roles in agriculture. *Streptomyces* spp. are well known as prolific producers of secondary metabolites with antimicrobial activity^26,27^, *Paenibacillus* spp. contribute to nutrient mobilization and plant growth promotion^28,29^, and *Pseudomonas* spp. are among the most established biocontrol agents^15,30,31^. All isolates reduced disease severity and promoted root development, particularly *Pi*, while pathogen abundance remained unchanged (Fig. 2A, B). This pattern is consistent with the SEM analysis presented in this study (Fig. 5A), which showed that inoculation treatment was the primary driver of microbiome structure and pathogen dynamics in the rhizosphere^6,10,32,33^. Besides, only inoculation with *Pi* significantly reduced bacterial alpha diversity, as indicated by both Shannon and Chao1 indices (Fig. 2C, D). Such reduction indicates that the strain altered rhizosphere community structure, resulting in lower richness and evenness and increased dominance of specific taxa. Notably, ASVs assigned to the inoculated genera represented a small fraction of total reads at the time of sampling (mean 0.5-1.0%, maximum 2.46%) (Fig. S4), indicating that these shifts were not driven by overrepresentation of the inoculant but rather by broader community restructuring. This pattern is consistent with previous reports of transient inoculant effects followed by a decline in relative abundance (after 15 days) due to interactions with native microbiota^34^.

The structural equation modeling (SEM) analysis provided further insight into the hierarchical relationships among inoculation (Fig. 5A), microbiome structure (PCoA1), pathogen abundance, and plant performance (root dry mass). The model indicated that inoculation treatment exerted a strong effect on microbiome structure and pathogen abundance, while disease severity was primarily explained by treatment effects rather than by direct changes in microbiome structure or pathogen load. Thus, the protective effect of the inoculants appears to be primarily driven by the inoculation treatment, which simultaneously influenced disease severity and reshaped the rhizosphere microbiome. In this context, although microbiome structure significantly influenced pathogen abundance, these changes did not translate into direct effects on disease severity, reinforcing the central role of inoculation treatment in determining plant health outcomes.

The combined 16S, ITS, and metagenomic data indicate that microbiome assembly was more strongly shaped by the inoculation of beneficial bacteria than by pathogen challenge, highlighting the dominant ecological filtering imposed by the introduced strains in the rhizosphere. While *Sv* induced pronounced shifts and *Po* triggered subtler restructuring, *Pi* reduced bacterial diversity yet produced communities distinct from other treatments, including when inoculated together with the pathogen. Moreover, all strains, when combined with *B. sorokiniana*, assembled communities distinct from bulk soil, control, and pathogen-only treatments, suggesting that inoculants redirect community assembly toward novel ecological states^35,36^.

Recent studies have shown that inoculation with beneficial bacterial strains not only directly influences plant performance but also shapes the structure, complexity, and function of the rhizosphere microbiome. For instance, Cunha et al.^37^ demonstrated that bacterial inoculation modulated soil chemical properties, altered plant gene expression, and enhanced microbial network complexity in a strain-specific manner closely linked to plant responses. Similarly, our findings are consistent with those reported in wheat by Nishisaka et al.^15^, where shifts in microbial networks were associated with increased resilience to pathogen attack, supporting the idea that inoculants can occupy and stabilize ecological niches disrupted by infection. Notably, this pattern is consistent with the concept of soil disease, whereby pathogen presence or even high abundance does not necessarily translate into disease incidence, as previously reported^7,15^. In this context, we further demonstrate that invasion of the rhizosphere by a fungal pathogen can actively reshape both the taxonomic structure and the functional potential of the resident microbiome, promoting community-level shifts that enhance pathogen suppression. Together, these findings reinforce the view that beneficial microorganisms function as ecological engineers, reorganizing microbial networks and metabolic capacities within the rhizosphere in ways that stabilize plant health despite pathogen pressure.

Regarding fungal communities, responses to the treatments were considerably more modest and less consistent than those observed for bacteria. Alpha diversity remained unchanged across treatments (Fig. S3), and only a limited number of significant pairwise differences emerged (Table S3), especially under *Sv*- and *Pi*-based treatments, indicating overall stability in fungal assemblages. Nevertheless, *Po*-based treatments showed stronger restructuring of fungal community composition (Fig. 4B). Outside this treatment, compositional shifts were scattered (e.g., differences between *B. sorokiniana* and *Sv* + *B. sorokiniana*), indicating that most fungal taxa were minimally affected by either pathogen infection or bacterial inoculation. Outside this treatment, compositional shifts were observed (e.g., differences between *B. sorokiniana* and *Sv* + *B. sorokiniana*), showing that most fungal taxa were minimally affected by either pathogen infection or bacterial inoculation. This subdued response is consistent with previous work showing that fungi often maintain functional and compositional stability despite fluctuations in bacterial communities, partly due to their slower turnover, extensive hyphal networks, and greater buffering capacity against short-term disturbances^37–39^. Consequently, while meaningful fungal shifts did occur, bacterial communities provided a clearer and more sensitive lens for interpreting treatment effects on rhizosphere microbiome assembly.

Despite the overall stability of fungal community assembly, the compositional shifts observed under *Po*-based treatments indicate that beneficial bacteria may also influence fungal assemblages through indirect ecological mechanisms. These cross-kingdom interactions may arise from altered rhizodeposition patterns, competition for shared resources, or the production of metabolites that affect fungal physiology and interactions^40,41^. Similar cross-domain restructuring has been reported in other crop systems, where PGPR inoculation altered fungal community composition and functional potential^37,42,43^. Thus, even though fungal beta diversity responded less strongly than bacterial communities, the compositional changes observed here suggest that beneficial inoculants can influence both bacterial and fungal components of the rhizosphere. To evaluate whether these community shifts translated into functional consequences for the rhizosphere, we then examined metagenomic indicators of microbial activity and resilience.

Pathogen infection was associated with reduced richness of plant growth-promoting (PGP) genes and a collapse in biosynthetic gene cluster (BGC) network complexity, reflective of an erosion in functional redundancy within the rhizosphere. Such functionally impoverished states mirror patterns observed in disease-conducive soils, where simplified microbial communities fail to resist invasion and maintain ecosystem functionality^10,12,13^. Furthermore, the decline in functional diversity and redundancy is a characteristic response to pathogen perturbation, with pathogen pressure undermining both taxonomic and functional resilience of the rhizosphere microbiome^44,45^. In contrast, inoculation with *Sv* and *Pi* restored PGP gene diversity and enriched functions related to stress tolerance, amino acid metabolism, and membrane transport, traits linked to plant resilience under biotic stress. Similar enhancements in rhizosphere microbial functional diversity following inoculation have been documented in other systems^37,46^.

Although inoculated plants exhibited reduced SPAD values (Fig. S2), this response does not necessarily conflict with the enrichment of genomic PGP potential observed in the rhizosphere microbiome (Fig. 7A). The observed variation in chlorophyll-related parameters may reflect transient physiological adjustments associated with microbial colonization, activation of plant metabolic pathways, or resource allocation trade-offs during plant–microbe interactions, rather than impaired plant performance. Moreover, the detection of PGP-related genes by genomic analyses represents functional potential that may not be fully expressed under in vivo conditions. Thus, reduced SPAD values should be interpreted within the broader context of conditional microbial trait expression and dynamic host physiological responses.

In this study, while *Sv* and *Pi* were primarily associated with functions linked to stress tolerance and secondary metabolism, *Po* + *B. sorokiniana* showed enrichment in genes related to redox balance, transcriptional regulation, and DNA transfer systems. These traits indicate a different mode of action, favoring cellular homeostasis and potential horizontal gene transfer, which may facilitate microbial adaptation and niche colonization under pathogen pressure^47,48^. Plus, enriched genes associated with protein turnover (*prc*) and chemotaxis (*cheY*, *swrC*) could potentially support adaptive responses to root-associated niches^49,50^. Protein turnover is critical for maintaining cellular homeostasis under stress^51^, while chemotaxis genes may enhance the ability of microbial populations to sense and colonize root exudate gradients^52^, thereby improving competitiveness and persistence in the rhizosphere^53^. In literature, for instance, inoculation with plant growth-promoting rhizobacteria (PGPR) has been shown to modulate both microbial structure and functional profiles, enhancing stress-associated functions^54^. More recently, Francoli et al.^55^ demonstrated that microbial consortia inoculation boosted genes encoding siderophore production, antimicrobial lipopeptides, and increased nutrient uptake (e.g., iron chelation and transporters), enhancing maize plant fitness. Simultaneously, BGC profiling revealed the recovery of NRPS, PKS, RiPP, and terpene clusters, reinstating metabolic versatility essential for antimicrobial production and microbial communication^56,57^. This coupling between PGP enrichment and biosynthetic recovery show that beneficial inoculants not only buffer the plant against disease but also preserve microbial community potential for secondary metabolism.

Taxonomic patterns also highlighted bacterial groups associated with disease outcomes. Random Forest analysis (Fig. 5C) identified several bacterial families, including Chitinophagaceae, Haliangiaceae, Myxococcaceae, and Xanthobacteraceae, as strong predictors of disease severity under pathogen challenge. Many members of these groups are known for their metabolic versatility and ecological roles in nutrient cycling, microbial predation, or antagonistic interactions, suggesting that specific components of the resident microbiome may contribute to differences in disease outcomes among pathogen-inoculated treatments^15,37,58^. Similar recruitment patterns under stress have been reported in wheat^15^ and common bean^59^ microbiome systems, suggesting that the restoration of ecological functionality may be underpinned by conserved host–microbe interactions. Plus, these taxonomic enrichments coincided with the recovery of both PGP genes and biosynthetic clusters, showing the role of microbial recruitment in reinstating community resilience.

Moreover, when all treatments were considered together, microbiome composition did not explain additional variation in disease severity beyond the effect of inoculation treatment, as indicated by the SEM analysis (Fig. 5A). However, when the analysis was restricted to pathogen-challenged treatments (pathogen and beneficial bacterial + pathogen treatments), Random Forest analysis showed that bacterial community composition explained a substantial proportion of the variation in disease severity (48%). These results suggest that although disease suppression is primarily driven by the inoculant treatment, differences in resident microbial communities may influence disease outcomes under pathogen pressure.

Taken together, our results highlight that targeted bacterial inoculants can reverse pathogen-induced perturbations in the rhizosphere by simultaneously protecting the plant, restructuring the microbiome, and restoring its biosynthetic and ecological complexity. Their protective effect comes not only from direct antagonism, but also from niche occupation, treatment-driven ecological restructuring, and the reinforcement of community-level functions.

Consistently, the three bacterial isolates, *Streptomyces virginiae* CMAA1738 (*Sv*), *Paenibacillus ottowii* CMAA1739 (*Po*), and *Pseudomonas inefficax* CMAA1741 (*Pi*), reduced disease severity and enhanced root growth while reshaping the rhizosphere microbiome under pathogen pressure. Notably, *Sv* and *Pi* reinstated plant growth-promoting gene diversity and biosynthetic network complexity, enriching functions related to stress tolerance, nutrient metabolism, and secondary metabolite production. Variation in disease severity among pathogen-inoculated treatments was associated with differences in bacterial community composition, highlighting the role of specific microbial groups in disease outcomes. Together, these results support the view that beneficial microbes act as ecological engineers, restoring functional resilience in the rhizosphere. Future work should assess the stability of these effects across soils and environments and explore the design of tailored microbial consortia for field-scale applications.

## Methods

### Bacteria isolation from wheat rhizosphere

The bacterial isolates were obtained from two wheat landraces, Karakilcik (Turkey) and Iran 1-29-11334 (Iran). Agricultural soil with a history of wheat cultivation was collected in Assis, SP (29°39’42” S, 50°24’44” O) and used to grow the wheat landraces in controlled conditions using a climate room. Rhizosphere sampling and bacterial isolation were performed according to Rossmann et al.^42^.

The rhizosphere soil from each cultivar was collected during the reproductive stage of the landrace’s plants, following the decimal codes 60–69 and 70–79 for cereal growth stages according to Zadoks et al.^60^. Then, 10 g of dry soil was diluted in 90 mL of 0.85% saline solution, forming the first dilution (10^–1^) of a serial dilution. Subsequently, 1 mL of this solution was added to 9 mL of saline solution, corresponding to a 10^–2^ dilution, and this process was repeated until reaching a 10^–5^ dilution. A volume of 100 μL from the 10^–3^, 10^–4^, and 10^–5^ dilutions was plated onto TSA (Tryptic Soy Agar; 1:10) using a Drigalski loop. Taking into account two plant genotypes (Karakilcik and Iran 1-29-11334), eight replicates, three dilutions (10^–3^, 10^–4^, and 10^–5^), and three plates per dilution, a total of 144 plates were inoculated and incubated at 28 °C for four days. After incubation, colony-forming units (CFUs) from the three rhizosphere soil dilutions were counted. Plates with colony numbers outside the acceptable precision and repeatability range (i.e., more than 300 or fewer than 30 CFUs) were discarded. From the remaining plates, one representative plate per sample was selected as the best representation of the microbial community. Isolation was performed using the streak plate technique, transferring colonies from the dilution plates to TSA plates. The isolates were then preserved using the glycerol method and stored at –80 °C.

### Pathogen growth inhibition

The fungal pathogens *Bipolaris sorokiniana* and *Fusarium graminearum* were subcultured on Potato Dextrose Agar (PDA) and incubated for five days. Bacterial isolates were prepared by inoculating a 20 μL aliquot from Castellani tubes into GY medium (10 g yeast extract, 15 g agar, 0.01 g FeSO_4_·H_2_O, 0.5 g KCl, 0.5 g MgSO_4_, 1 g K_2_HPO_4_, and 10 g dextrose per 1 L of distilled water) and incubated for 24 h.

In the initial screening, a PDA plate was inoculated with a fungal disc at the center and four different bacterial isolates positioned equidistant around it. Isolates that exhibited antifungal activity were subsequently tested individually in triplicate. Fungal growth inhibition was assessed by measuring the inhibition zone diameter, fungal colony diameter, and overall fungal growth.

### Production of indole acetic acid

The bacterial isolates were evaluated for indole-3-acetic acid (IAA) production through an initial qualitative assay, followed by a quantitative colorimetric analysis adapted from Quecine^61^ for selected strains. In the experiment setup, isolates were inoculated into 10% tryptic soy broth (TSB) supplemented with 5 mM L-tryptophan and incubated in the dark at 28 °C for 72 h under constant agitation.

After incubation, cultures were centrifuged at 10,000 × *g* for 5 min, and 900 μL of the supernatant was transferred to cuvettes containing 400 μL of Salkowski reagent^62^. Samples, prepared in triplicate, were incubated in the dark at room temperature for 2 h before absorbance measurements were taken at 520 nm using a spectrophotometer. IAA quantification was normalized against a standard curve prepared with commercial IAA. A previously characterized IAA-producing bacterial strain from the Embrapa Environment Microorganism Collection was used as a positive control, while uninoculated medium was included as a negative control.

### Phosphate solubilization activity

The isolates were streaked on GY medium and incubated at 28 °C for 48 h, followed by qualitative screening for phosphate solubilization on solid NBRIP medium^63^, composed of 10 g of C_6_H_12_O_6_, 5 g of MgCl_2_·6H_2_O, 0.25 g of MgSO_4_·7H_2_O, 0.2 g of KCl, 0.1 g of (NH_4_)2SO_4_, 5 g of Ca_3_(PO_4_)_2_, and 15 g of agar per liter of distilled water. Plates were prepared in triplicate and incubated at 28 °C for 7 days to allow halo formation. Isolates producing clear halos around colonies were considered capable of phosphate solubilization. The *Bacillus* sp. strain LMA42, formerly identified as *Bacillus aryabhattai* and known for its phosphate-solubilizing capacity^64^, was obtained from the Embrapa Environment Microorganism Collection and used as a positive control. In addition, an uninoculated sterile medium was included as a negative control to account for potential abiotic phosphate solubilization.

### Nitrogen fixation activity

The nitrogen fixation test was performed according to the Dobereiner method^65^, following an initial screening for other beneficial traits. For this, the bacteria were inoculated onto a semi-solid NFB medium containing 5 g of malic acid, 0.5 g of K_2_HPO_4_, 0.2 g of MgSO_4_·7H_2_O, 0.1 g of NaCl, 0.01 g of CaCl_2_·2H_2_O, 4 mL of Fe-EDTA (1.64% solution), 2 mL of bromothymol blue (0.5%), 2 mL of micronutrient solution (0.2 g L^–1^ of Na_2_MoO_4_·2H_2_O, 0.235 g L^–1^ of MnSO_4_·H_2_O, 0.28 g L^–1^ of H_3_BO_3_, 0.008 g L^–1^ of CuSO_4_·5H_2_O), 1.8 g of agar, dissolved in 1 L of distilled water with the pH adjusted to 6.5. After incubation at 28 °C for five days, positive results were indicated by a change in the color of the culture medium and the formation of a characteristic pellicle. The experiments were performed in triplicate, and a strain of *Azospirillum brasilense* strain V5-b6^66^, provided by Embrapa Agrobiology, was used as a positive control. In addition, an uninoculated sterile medium was included as a negative control to account for any spontaneous color changes.

### Bacterial inoculum preparation and seed treatment

The three isolated and selected antagonist bacteria, *Streptomyces virginiae* strain CMAA1738 (*Sv*), *Paenibacillus ottowii* strain CMAA1739 (*Po*), and *Pseudomonas inefficax* strain CMAA1741 (*Pi*), were initially cultivated on agar media following the protocol described by Nishisaka et al.^25^. Subsequently, for inoculum preparation, each bacterial strain was grown in Tryptic Soy Broth (TSB) liquid medium under agitation at 160 rpm, at 28 °C, for 24 to 48 h. To obtain the bacterial pellet, the cultivated bacteria were centrifuged at 8000 × *g* for 5 min. The bacterial cells were then thoroughly resuspended in 20 mL of 0.85% saline solution. The inoculum concentration was determined by UV spectrophotometry (OD₅₅₀), where an OD₅₅₀ of 0.1 corresponded to 10^6^ CFU mL⁻¹ for *Sv*, 10^8^ CFU mL⁻¹ for *Po* and *Pi*, as determined by plating. The same inoculum concentrations were used for both seed inoculation and booster applications.

Seed disinfection and treatment was performed on the same day as sowing. Wheat seeds were surface-disinfected by immersion in 0.5% sodium hypochlorite for 2 min, three rinses in sterile distilled water, followed by 70% ethanol (1 min), and three subsequent rinses in sterile distilled water, to remove any residual disinfectants. Subsequently, 20 g of wheat seeds were immersed in 20 mL of each bacterial suspension, corresponding to a seed-to-liquid ratio of 1:1 (w/v), and subjected to constant agitation at 160 rpm for 1 h to ensure uniform inoculum distribution and homogeneous coating of the seeds prior to sowing. The control treatment followed the same protocol, but ultrapure autoclaved water was used instead of the bacterial suspension. After treatment, the seeds were immediately planted according to their respective treatments.

### *Bipolaris sorokiniana* inoculum preparation

The wheat pathogenic fungus *Bipolaris sorokiniana* was first grown on Potato Dextrose Agar (PDA) medium and incubated at 23 °C for 7 to 15 days. To harvest fungal conidia, 10 mL of a 0.8% Tween solution (Tween 80) was added to each plate, and the conidia were collected. The resulting suspension was thoroughly mixed, and conidia concentration was determined using a Neubauer chamber. For plant infection, 1 mL of the fungal suspension, standardized to 10^4^ conidia per mL in 0.8% Tween solution, was applied to the base of each plant stem.

### Plant bioassay and experimental design

The bioassay was conducted under controlled conditions in a climate room using soil collected from the experimental field of Embrapa Environment (22°72'77.11” N, 47°01'76.85” E), classified as Oxisol. The soil was dried and sieved following the technical guidelines provided by Embrapa Wheat^67^. Base saturation was adjusted to 70%, as recommended for wheat varieties sensitive to soil acidity, according to Boletim 100 of the Agronomic Institute of Campinas (IAC)^68^. The soil was then incubated for 15 days before the experiment. Field soil sampling was conducted with authorization and registration in the Brazilian National System for the Management of Genetic Heritage and Associated Traditional Knowledge (SISGen) under registration number A5EB05F.

The experiment used 250 mL pots, each containing 200 g of dried and sieved soil, under nine treatments encompassing both plant-associated and bulk soil conditions. Plant-associated treatments included infection with *Bipolaris sorokiniana*, *Streptomyces virginiae* strain CMAA1738 (*Sv*), *Paenibacillus ottowii* strain CMAA1739 (*Po*), and *Pseudomonas inefficax* strain CMAA1741 (*Pi*), either individually or in combination (e.g., *Bipolaris sorokiniana* + beneficial bacteria). The experimental treatments included: (i) bulk soil; and plants inoculated with (ii) water treatment (control); (iii) *B. sorokiniana* alone, (iv) *S. virginiae* strain CMAA1738 (*Sv*) alone, (v) *P. ottowii* strain CMAA1739 (*Po*) alone, (vi) *P. inefficax* strain CMAA1741 (*Pi*) alone, (vii) *Sv* + *B. sorokiniana*, (viii) *Po* + *B. sorokiniana*, and (ix) *Pi* + *B. sorokiniana*. Each treatment comprised six replicates (54 pots in total). Fifteen seeds were initially sown per pot and later thinned to 10 plants per pot.

Wheat cultivar BRS Guamirim, which is susceptible to *B. sorokiniana*^38^, was used for the antagonistic bioassay. Seven days after sowing bacterial-treated seeds, plants designated for pathogen challenge were inoculated with 1 mL of *B. sorokiniana* conidia suspension (10^4 ^mL^–1^). Fifteen days after sowing and 8 days post-pathogen infection, a bacterial boost dose was applied. The boost dose consisted of 1 mL of the bacterial suspension applied directly to the stem base of each plant (10 plants per pot), totaling 10 mL per pot. By the end of the experiment, on the 35th day, the following parameters were evaluated: Disease Severity Index (DSI), plant height, fresh biomass, and relative chlorophyll index. The experiment was conducted under controlled environmental conditions (21 ± 2°C, 12-hour light/dark photoperiod). Soil moisture was adjusted according to plant development stages, ranging from 10 to 20% (v/w)^38^.

### Disease severity index calculation

For disease severity index calculation, the proportion of infected plants was evaluated and classified into four categories: asymptomatic (0 = plants with no visible symptoms), mild symptoms (1 = plants with a slight dark lesion limited to the cotyledon leaf), moderate symptoms (2 = plants with dark or reddish moderate lesions on the stem), and severe symptoms (3 = plants with dark, severe lesions extending above the first leaf or plants that were dead)^38^. Following classification, the Disease Severity Index (DSI) was calculated for each pot using a method adapted from McMillan et al.^69^. The DSI was determined with the formula: (1 x percentage of plants in category 1) + (2 x percentage of plants in category 2) + (3 x percentage of plants in category 3), divided by the total number of categories (3). The maximum possible DSI was set at 100%^38^.

### Measurements of the plant physiological parameters

At the end of the experiment, plant height, as well as fresh and dry biomass, were measured. Height was recorded from the base of the plant to the tip of the tallest leaf using a ruler (±0.5 cm), with measurements taken from five plants per pot to calculate the average. Roots were carefully washed and trimmed, separating the shoot and root portions for subsequent analysis. Fresh biomass (shoots and roots) was weighed using a semi-analytical balance (±0.01 g). The shoots and roots were then placed in separate paper bags and dried in an oven at 65 °C for 7 days. After drying, the plant material was weighed again using a semi-analytical balance (±0.01 g) to determine the dry biomass for each treatment.

The relative chlorophyll content (SPAD index) was estimated non-destructively using a SPAD-502 chlorophyll meter (Minolta Corp., Ramsey, NJ, USA). Measurements were taken at the midpoint of the leaf blade on the youngest fully expanded leaf of each plant to ensure consistency. Five leaves per pot were measured (one per plant), and the average SPAD value per pot was used for statistical analysis.

### Soil sampling, DNA isolation and sequencing

Rhizosphere soil sampling was performed by carefully removing all plants from each pot and gently shaking the roots to dislodge loosely attached soil. The soil that remained adhered to the roots, defined as rhizosphere soil, was collected. For each biological replicate, rhizosphere soil was pooled from the roots of all 10 plants within the same pot, generating one composite sample per pot. The collected material was transferred into 1.5 mL microtubes and stored at −20 °C until further molecular analyses. Samples from bulk soil treatments were also collected and stored in the freezer. Total genomic DNA extraction was carried out using the DNeasy PowerSoil® Kit (QIAGEN, catalog #12888-50) according to the manufacturer’s protocol. The quality and concentration of the extracted DNA were assessed using a NanoDrop® ND-2000 Spectrophotometer and a QUBIT® 2.0 Fluorometer (Thermo Fisher Scientific, Wilmington, DE, USA), respectively.

Metataxonomic sequencing targeted the V4 region of the bacterial 16S rRNA gene and the ITS1 region of the fungal ITS. For that, DNA samples were lyophilized and sent to Argonne National Laboratory (Lemont, IL, USA) for library preparation and sequencing using the Miseq-Illumina platform. For the V4 region of the 16S rRNA gene, the primer pair 515 F (5’-GTGYCAGCMGCCGCGGTAA-3’) and 806 R (5’-GGACTACNVGGGTWTCTAAT-3’) was utilized^70,71^. Similarly, for the ITS1 region of the fungal ITS, the primer pair ITS1f (5’-CTTGGTCATTTAGAGGAAGTAA-3’) and ITS2 (5’-GCTGCGTTCTTCATCGATGC-3’) was employed^72^.

For metagenomic sequencing, we selected samples from the control treatment (seed inoculated with water), the *B. sorokiniana*-inoculated plants, and each of the three treatments combining *B. sorokiniana* with one of the antagonistic bacteria, totaling 30 samples (5 treatments x 6 replicates). These treatments were chosen as the most relevant to our research objectives. The selected samples were submitted to Novogene Corporation Inc. (El Monte, California, USA) and sequenced on the Illumina NovaSeq 6000 platform, generating 150 bp paired-end reads.

### *Bipolaris sorokiniana* gene *cos*A quantification by qPCR

Quantitative polymerase chain reaction (qPCR) was used to quantify the *B. sorokiniana*-specific *cos*A gene in the sampled soil, employing the primer pair *Cos*A_F_519 (5’ TCAAGCTGACCAAATCACCTTC 3’) and *Cos*A_R_248 (5’ AATGTCGAGCTTGCCAAAGT 3’)^73,74^. The qPCR reaction had a final volume of 10 μL, including 5 μL of SYBR Green ROX qPCR (Thermo Fisher Scientific, Middletown, VA, USA), 1 μL of each primer (final concentration of 2 μM), 2 μL of template, and 2 μL of ultrapure water (Milli-Q). Absolute quantification of the *cos*A gene was performed using a standard curve generated from a four-point ten-fold serial dilution (1:10), in triplicate, of purified genomic DNA from *Bipolaris sorokiniana* strain BS0208. The dynamic range of the curve spanned from 3.90 × 10^10^ to 3.90 × 10^2^ gene copies (based on 2 µL of template per reaction). Four dilution points were used for linear regression analysis. The standard curve exhibited high linearity (R^2^ = 1.00) with a slope of −3.32, corresponding to an amplification efficiency of 99.98%. Quantification experiments were performed using a ViiA 7 Real-Time PCR System (Figure. S5).

### Statistical and bioinformatic analysis

The averages of plant height, Disease Severity Index (DSI%), fresh and dry biomass (shoots and roots), chlorophyll content, and *cos*A gene copy number obtained by qPCR were compared using Scott-Knott test to determine significant differences between treatments (*p* < 0.05). Prior to analysis, data were tested for normality using the Shapiro-Wilk test, and homogeneity of variances was assessed to ensure that the assumptions of parametric analysis were met. Statistical analyses were performed using RStudio software, version 2024.12.0. All results are presented as mean ± standard error.

For amplicon sequencing analysis (metataxonomic), all reads were processed and assembled using DADA2 version 1.24.0^75^. Primer sequences were removed with Cutadapt version 5.0^76^. Subsequently, quality control measures were applied to filter out low-quality reads (Q20 or lower). Taxonomic classification was conducted using the Silva database (version 138.1) for bacterial reads and the UNITE database (version 9.0) for fungal reads^77–81^. The exploratory analysis included alpha diversity (Chao1 and Shannon indices) and beta diversity (Bray-Curtis distance), visualized through Principal Coordinate Analysis (PCoA). Besides, a structural equation modeling (SEM) was used to evaluate the causal relationships linking bacterial inoculation, microbiome structure, pathogen abundance, disease severity, and plant performance. The first axis of the bacterial community PCoA (PCoA1) was used as a proxy for microbiome structure. Pathogen abundance was represented by the log-transformed qPCR quantification of *Bipolaris sorokiniana*. SEM was implemented using the piecewise approach with the R package piecewiseSEM (v. 2.3.1)^82^. Four linear models were constructed to represent the hypothesized causal pathways: (i) microbiome structure (PCoA1) as a function of inoculation treatment; (ii) pathogen abundance as a function of microbiome structure and treatment; (iii) disease severity index (DSI) as a function of pathogen abundance, microbiome structure, and treatment; (iv) root dry mass as a function of disease severity and microbiome structure. Model fit was evaluated using the Fisher’s C statistic and the chi-squared test of directed separation. Standardized path coefficients were extracted for continuous variables. Total standardized effects were calculated by combining direct and indirect paths within the causal network.

Genome analysis was conducted using genome assemblies provided by our previous study, publicly available in NCBI: GCF_035853655.1 (*Sv*), GCF_035853635.1 (*Po*), and GCF_035853745.1 (*Pi*)^25^. Plant growth-promoting (PGP) genes were annotated using PGPg_finder v1.1.0 against the PLaBAse database^83,84^ using default parameters, including a minimum amino acid identity threshold of 50% for DIAMOND alignments. Biosynthetic gene clusters (BGCs) were identified with the antiSMASH web server, v.7.1.0^85^, and circular genome visualization was performed using the Genovi pipeline v.0.2.16^86^.

For shotgun metagenomic analysis, raw reads were assessed using FastQC v.0.12.1 and trimmed using Trimmomatic v.0.39^87^, retaining sequences with Q > 20. Metagenomic assembly was carried out using MEGAHIT v.1.2.9^88^ with default parameters. Contig coverage was calculated using BWA-MEM v.0.7.17 and SAMtools v.1.2. Taxonomic classification of contigs was performed with Kraken2 v.2.1.3^89^, using the RefSeq complete genome database.

PGP genes in the metagenomes were annotated using PGPg_finder against the mg-PLaBAse database^83,84,90^ using a minimum amino acid identity threshold of 50% for DIAMOND and a sequence length of 100 bp. Due to the compositional and high-dimensional nature of the data, outlier samples were identified based on community composition using distance to group centroid (betadisper), leave-one-out PERMANOVA influence (LOO-PERMANOVA), and ordination inspection. Samples deviating from group structure and disproportionately influencing PERMANOVA results were excluded from downstream analyses^91–93^. Alpha (richness and Shannon indices) and beta diversity (PCoA using Bray-Curtis) analyses were conducted on the annotated gene profiles. For functional comparison, a Kruskal-Wallis test (KW) was performed using the Level 3 (Lv3) functional categories from PLaBAse across all treatments (*p* < 0.05). Pairwise comparisons were performed using Welch’s t-test within STAMP v2.1.3^94^, specifically comparing *B. sorokiniana*-inoculated samples with those co-inoculated with selected isolates, using Level 5 (Lv5) PLaBAse gene annotations.

Biosynthetic gene clusters in the metagenomes were predicted using antiSMASH v.7.1.0, and BGC networking was performed using BiG-SCAPE v.1.1.2^95^. Results were visualized in Gephi v.0.10.1, where node size represented contig coverage and node color represented BGC class. BGC taxonomy was inferred based on contig classification and antiSMASH annotations. The significance of differences in BGC category coverage was assessed using the Kruskal-Wallis test (*p* < 0.05), and pairwise comparisons across treatments were performed using the Wilcoxon rank-sum test (*p* < 0.05).

## Supplementary information

41522_2026_1021_MOESM1_ESM

## Data Availability

All sequencing data generated in this study have been deposited in the NCBI Sequence Read Archive (SRA) under accession codes PRJNA1266611 and PRJNA1236363.
