# Supplementary material for: Bacterial inoculation drives microbiome-mediated resistance to a soil-borne pathogen in wheat
Source: NPJ Biofilms Microbiomes. 2026 May 26;12:160. doi: 10.1038/s41522-026-01021-8 (PMC13482813; doi:10.1038/s41522-026-01021-8)
Supplement: Supplementary file 1 [file 41522_2026_1021_MOESM1_ESM.pdf]

Bacterial inoculation drives microbiome-mediated resistance to a soil-borne pathogen in wheat

Caroline Sayuri Nishisaka[1,2†], Hélio Danilo Quevedo[1,3†], Thierry Alexandre Pellegrinetti[2], Fernanda de Almeida Godoy[1,3], Maike Rossmann[2], Lucas William Mendes[2], Rodrigo Mendes[1,3*]

[1] Embrapa Environment, Jaguariúna, SP, Brazil.

[2] Center for Nuclear Energy in Agriculture, University of São Paulo, Piracicaba, SP, Brazil.

[3] College of Agriculture “Luiz de Queiroz”, University of São Paulo, Piracicaba, SP, Brazil.

[†] These authors have contributed equally to this work and share the first authorship

**Corresponding author*: rodrigo.mendes@embrapa.br

This Supplementary Material contains Figures S1–S5 and Tables S1–S3.

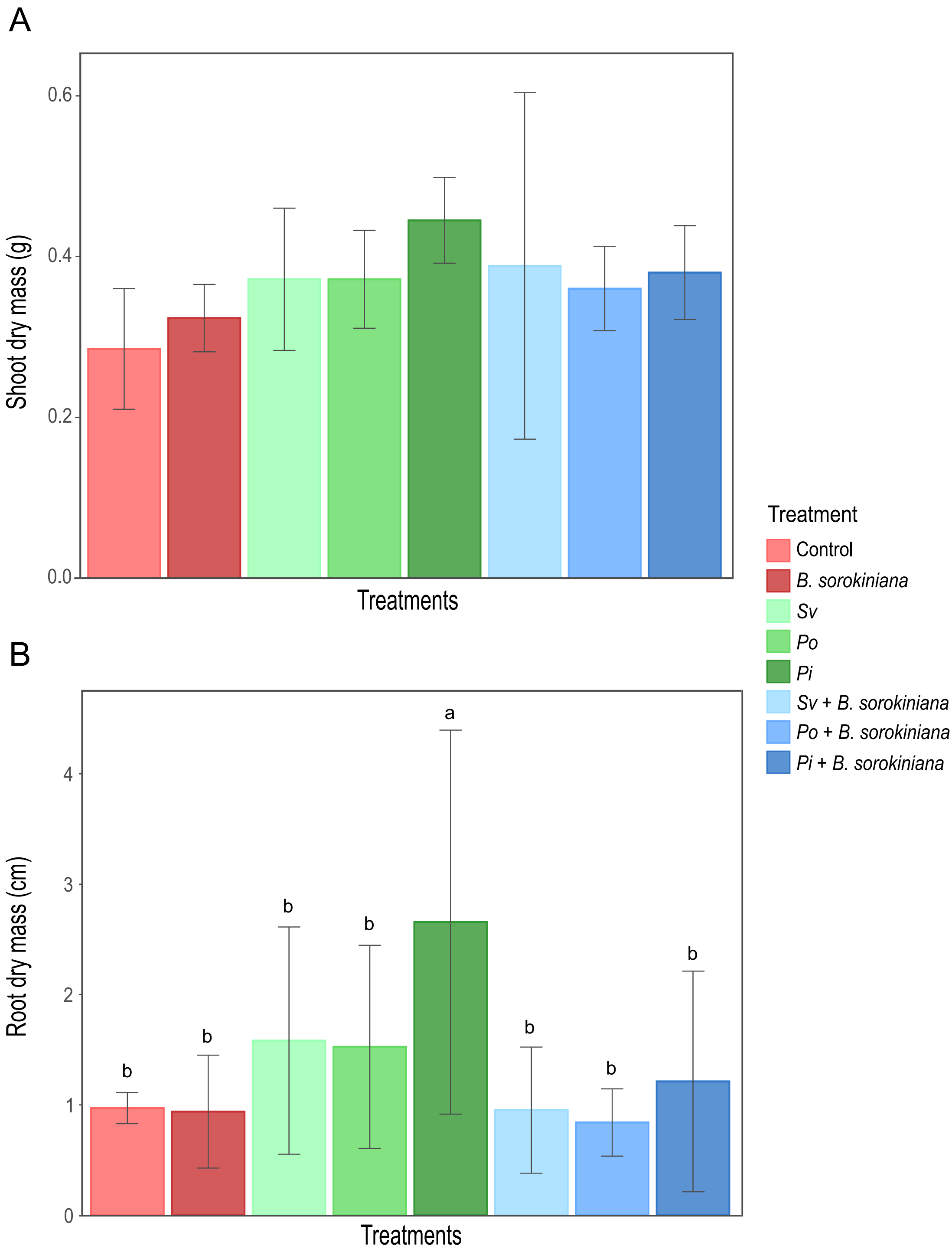

Figure S1. Shoot and root dry masses of wheat plants under different treatments. **A** Shoot dry mass. **B** Root dry mass. Bars represent mean values ± standard deviation. Different letters indicate significant differences among treatments according to Kruskal–Wallis test ($p < 0.05$). Control (non-treated plants), *B. sorokiniana* (plants inoculated with the pathogen *Bipolaris sorokiniana*), *Sv* (plants inoculated with *Streptomyces virginiae* strain CMAA1738), *Po* (plants inoculated with *Paenibacillus ottowii* strain CMAA1739), *Pi* (plants inoculated with the *Pseudomonas inefficax* strain CMAA1741), *Sv* + *B. sorokiniana* (plants inoculated with the *S. virginiae* and with *B. sorokiniana*), *Po* + *B. sorokiniana* (plants inoculated with the *P. ottowii* and with *B. sorokiniana*), and *Pi* + *B. sorokiniana* (plants inoculated with the *P. inefficax* and with *B. sorokiniana*).

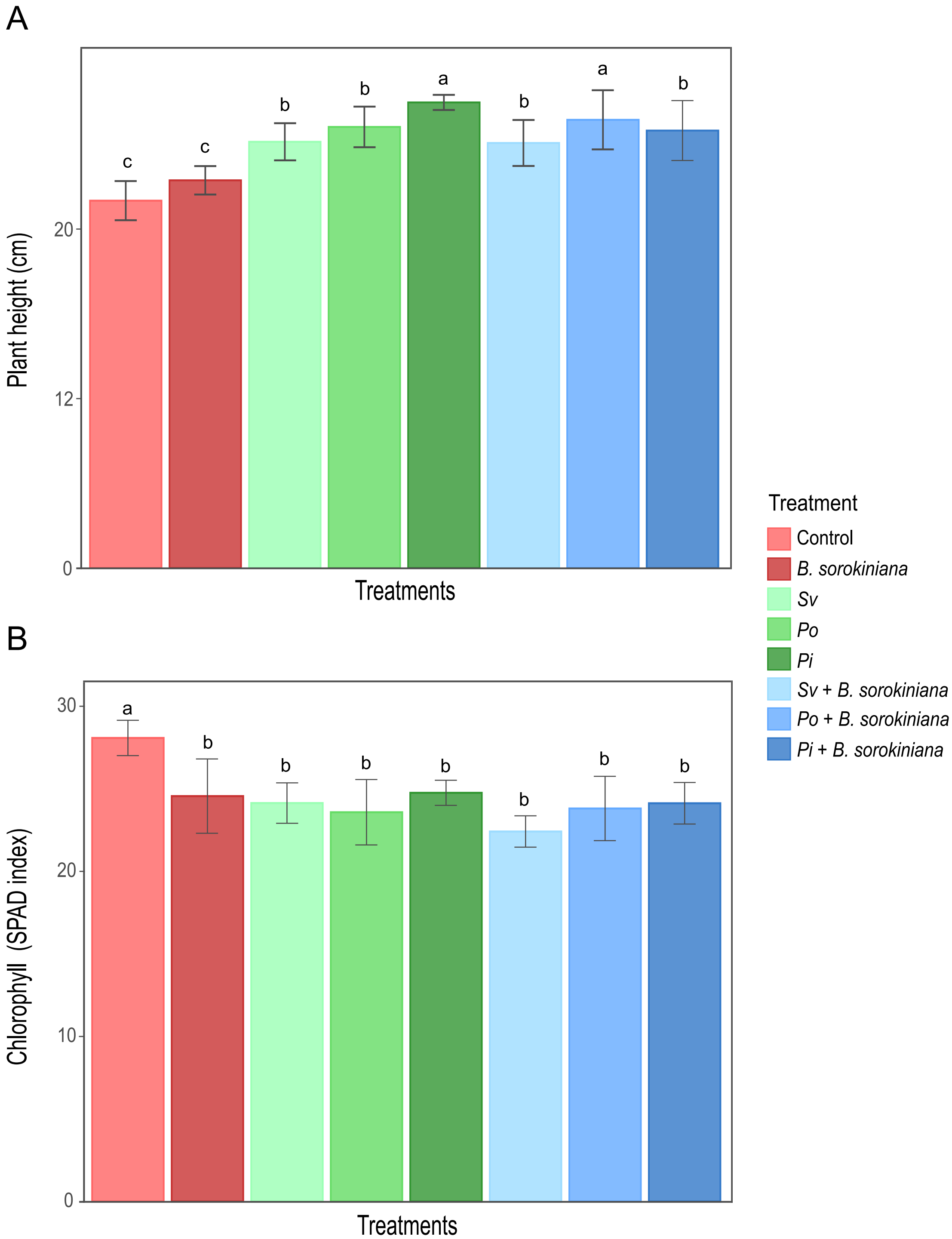

Figure S2. Plant height and chlorophyll content of wheat plants under different treatments. **A** Plant height. **B** Chlorophyll content (SPAD units). Bars represent mean values ± standard deviation. Different letters indicate significant differences among treatments according to Kruskal–Wallis test (p < 0.05). Control (non-treated plants), *B. sorokiniana* (plants inoculated with the pathogen *Bipolaris sorokiniana*), *Sv* (plants inoculated with *Streptomyces virginiae* strain CMAA1738), *Po* (plants inoculated with *Paenibacillus ottowii* strain CMAA1739), *Pi* (plants inoculated with the *Pseudomonas inefficax* strain CMAA1741), *Sv* + *B. sorokiniana* (plants inoculated with the *S. virginiae* and with *B. sorokiniana*), *Po* + *B. sorokiniana* (plants inoculated with the *P. ottowii* and with *B. sorokiniana*), and *Pi* + *B. sorokiniana* (plants inoculated with the *P. inefficax* and with *B. sorokiniana*).

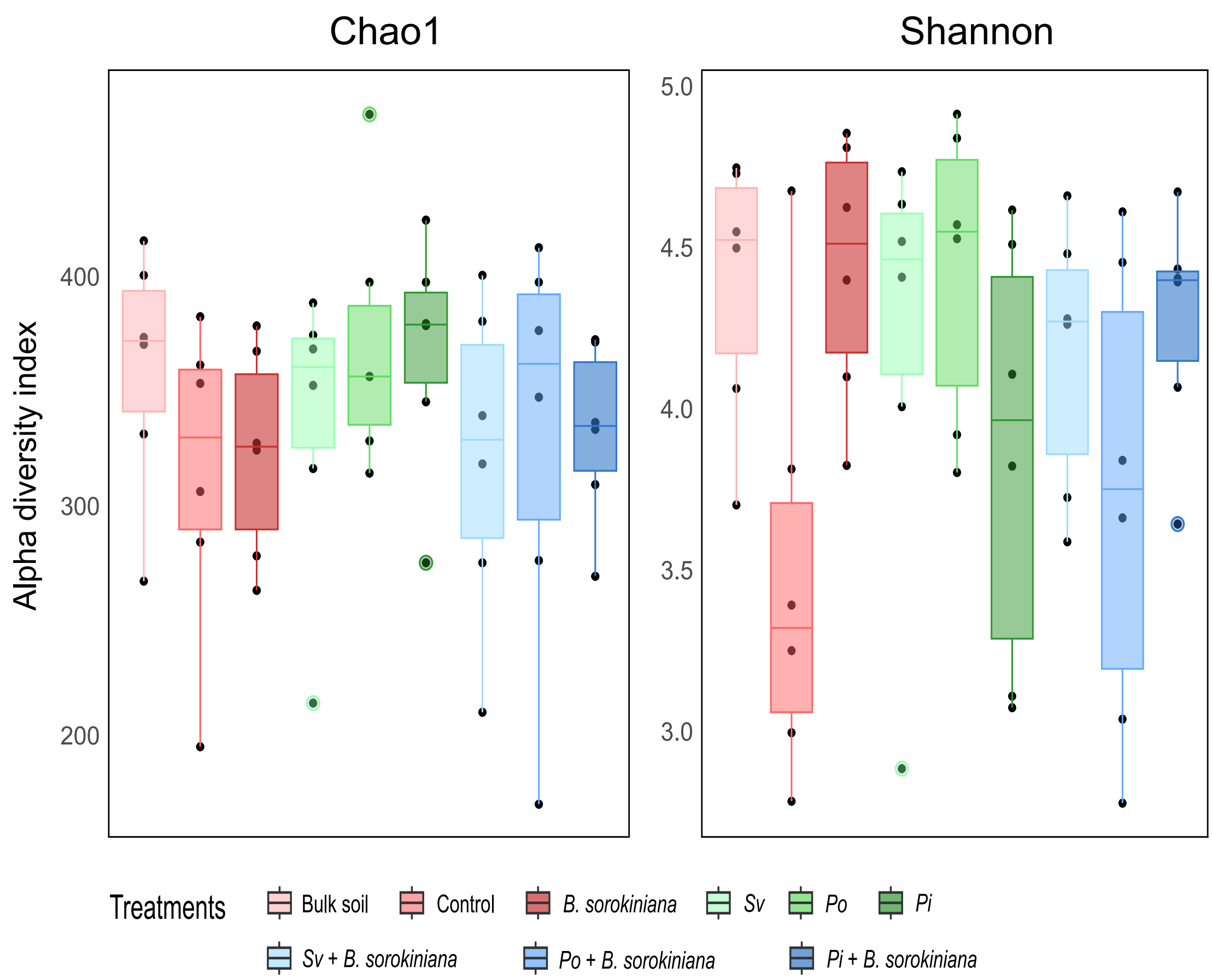

Figure S3. Fungal alpha diversity indices of wheat rhizosphere communities under different treatments. Chao1 richness and Shannon diversity are shown. No significant differences were detected among treatments (Kruskal–Wallis test, $p > 0.05$). Bulk soil (soil without plants), Control (non-treated plants), *B. sorokiniana* (plants inoculated with the pathogen *Bipolaris sorokiniana*), *Sv* (plants inoculated with *Streptomyces virginiae* strain CMAA1738), *Po* (plants inoculated with *Paenibacillus ottowii* strain CMAA1739), *Pi* (plants inoculated with the *Pseudomonas inefficax* strain CMAA1741), *Sv* + *B. sorokiniana* (plants inoculated with the *S. virginiae* and with *B. sorokiniana*), *Po* + *B. sorokiniana* (plants inoculated with the *P. ottowii* and with *B. sorokiniana*), and *Pi* + *B. sorokiniana* (plants inoculated with the *P. inefficax* and with *B. sorokiniana*).

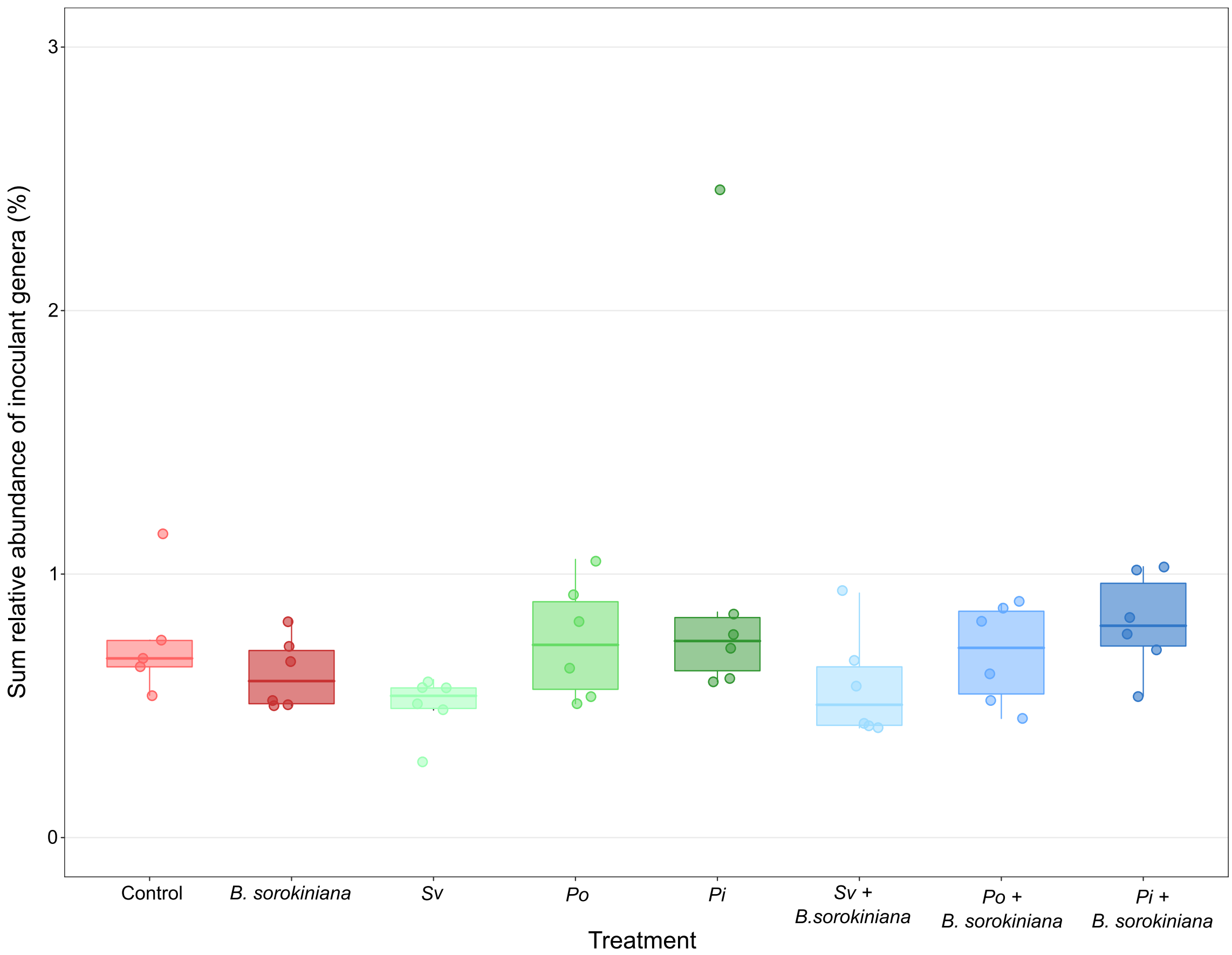

Figure S4. Cumulative relative abundance (%) of ASVs assigned to the inoculated genera (*Streptomyces*, *Paenibacillus*, and *Pseudomonas*) across treatments at the time of rhizosphere sampling (35 days after sowing; 20 days after the boost application). The inoculant-related genera accounted for < 2.5% of total reads in all cases.

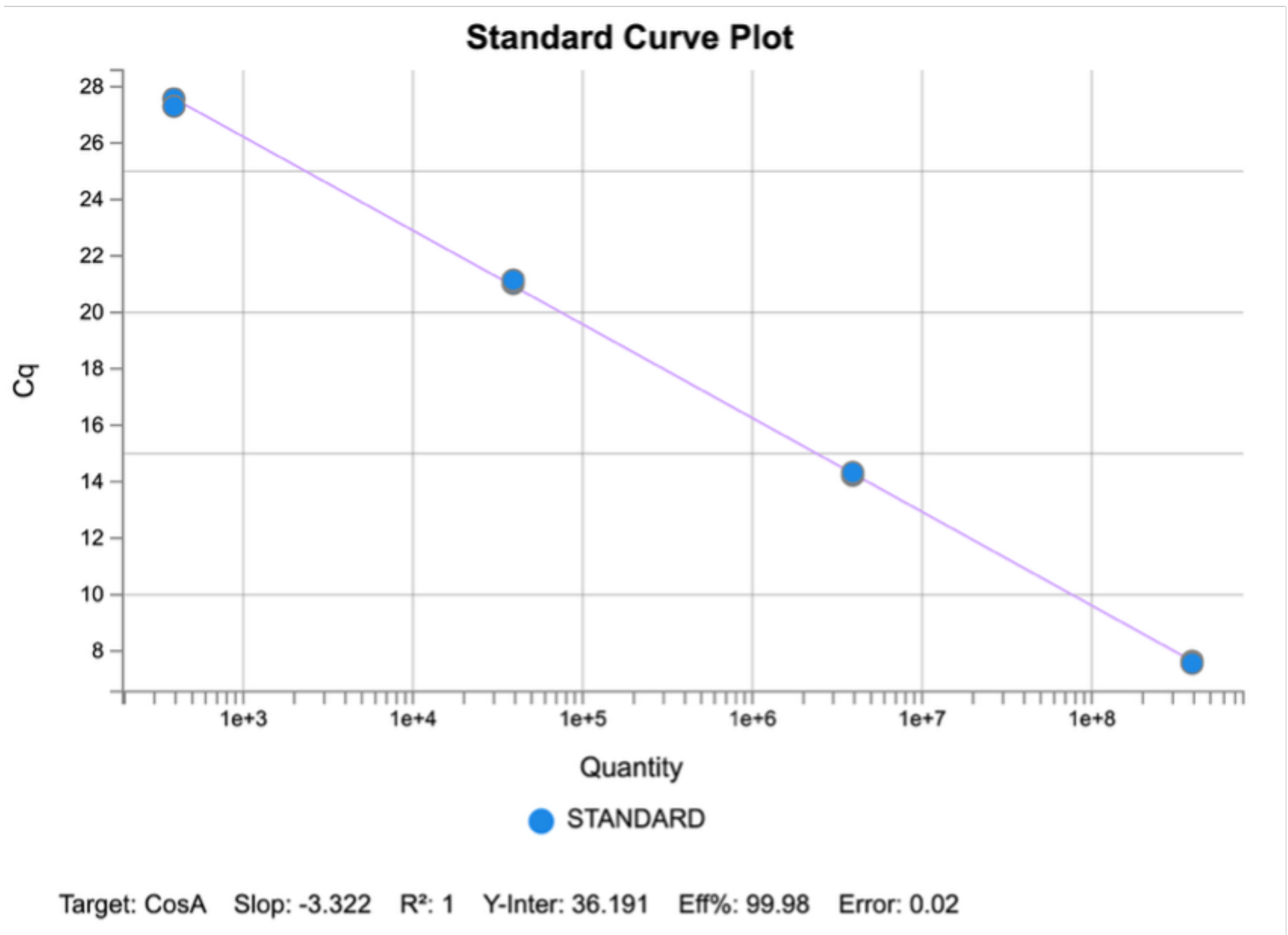

Figure S5. Standard curve used for absolute quantification of the *cos*A gene by qPCR. The curve was generated from a four-point ten-fold serial dilution series (1:10), performed in triplicate, using purified genomic DNA from *Bipolaris sorokiniana* strain BS0208. The dynamic range spanned from 3.90 x 10^{10} to 3.90 x 10^{2} gene copies per reaction (2 µL of template DNA). Linear regression analysis showed high linearity (R^2 = 1.00) with a slope of -3.32, corresponding to an amplification efficiency of 99.98%. Quantification assays were performed using a ViiA 7 Real-Time PCR System.

Table S1. Screening of beneficial traits and genetic characterization of bacterial isolates from ancestral and modern wheat genotypes. Taxonomic assignment was initially obtained from amplicon sequencing prediction.

Strain	Wheat cultivar	Bacterial species	IAA prod.	P solub.	N fix.	Antagonism to *B. sorokiniana*
W2_4/6	Turkey, Karakilcik	*Agrobacterium salinitolerans*	+	+	-	+
C5_3/14	Brazil, BRS Guamirim	*Agrobacterium salinitolerans*	+	+	-	-
C6_5/4	Brazil, Quartzo	*Bacillus altudinis*	NT	+	-	-
C6_2/2	Brazil, Quartzo	*Bacillus altitudinis*	+	+	+	+
C6_2/3	Brazil, Quartzo	*Bacillus altitudinis*	+	+	-	-
W1_3/8	Iran, 1-29-11334	*Bacillus cereus*	-	+	-	-
W2_3/14a	Turkey, Karakilcik	*Bacillus cereus*	+	-	-	-
C5_4/6	Brazil, BRS Guamirim	*Bacillus cereus*	-	+	-	-
W1_5/3	Iran, 1-29-11334	*Bacillus dentrensis*	NT	NT	-	-
C5_5/x	Brazil, BRS Guamirim	*Bacillus megaterium*	NT	-	-	NT
CMAA1743	Iran, 1-29-11334	*Bacillus megaterium*	+	+	+	+
C5_3/20	Brazil, BRS Guamirim	*Bacillus megaterium*	NT	+	-	-
W1_3/9	Iran, 1-29-11334	*Bacillus megaterium*	+	-	-	-
C5_3/14b	Brazil, BRS Guamirim	*Bacillus megaterium*	+	+	-	-
C6_2/12	Brazil, Quartzo	*Bacillus megaterium*	+	+	-	-
W2_5/bac	Turkey, Karakilcik	*Bacillus pseudomycoides*	NT	-	-	-
W2_2/bac	Turkey, Karakilcik	*Bacillus pseudomycoides*	NT	-	-	-
C6_5/bac	Brazil, Quartzo	*Bacillus pseudomycoides*	NT	-	-	-
W2_4/bac	Turkey, Karakilcik	*Bacillus pseudomycoides*	NT	-	-	+
C5_3/7	Brazil, BRS Guamirim	*Bacillus pseudomycoides*	NT	NT	-	-
C6_3/bac	Brazil, Quartzo	*Bacillus pseudomycoides*	NT	-	-	+
C6_2/bac	Brazil, Quartzo	*Bacillus pseudomycoides*	NT	-	-	-
C6_3/12	Brazil, Quartzo	*Bacillus proteolitycus*	-	+	-	-
C6_2/1b	Brazil, Quartzo	*Bacillus safensis*	+	-	-	+
W2_4/11	Turkey, Karakilcik	*Bacillus thuringiensis*	-	+	-	+
W1_2/bac	Iran, 1-29-11334	*Bacillus thuringiensis*	NT	-	-	-
W1_2/7	Iran, 1-29-11334	*Cupriavidus gilardii*	NT	NT	-	-
W2_2/anti8	Turkey, Karakilcik	*Gordonia terrae*	+	-	-	-
C5_3/13	Brazil, BRS Guamirim	*Gordonia terrae*	+	+	-	-
W2_2/10	Turkey, Karakilcik	*Lysinibacillus fusiformis*	+	-	-	-
C5_3/23	Brazil, BRS Guamirim	*Lysinibacillus fusiformis*	+	-	-	-
C6_4/7	Brazil, Quartzo	*Lysinibacillus fusiformis*	NT	+	-	NT
C6_4/8	Brazil, Quartzo	*Lysinibacillus fusiformis*	NT	-	-	-
C6_4/2	Brazil, Quartzo	*Lysinibacillus fusiformis*	NT	-	-	-
W1_2/4	Iran, 1-29-11334	*Lysinibacillus fusiformis*	+	-	-	NT
CMAA1742	Turkey, Karakilcik	*Lysinibacillus fusiformis*	+	+	+	+
C5_3/9	Brazil, BRS Guamirim	*Lysinibacillus fusiformis*	+	-	-	-
C6_2/4	Brazil, Quartzo	*Lysinibacillus fusiformis*	+	-	-	-
C5_3/19	Brazil, BRS Guamirim	*Lysinibacillus fusiformis*	NT	+	-	-
C5_4/10	BRS Guamirim	*Lysinibacillus fusiformis*	+	+	-	-
C6_4/9	Brazil, Quartzo	*Lysinibacillus pakistanensis*	+	+	-	-
C5_2/8	Brazil, BRS Guamirim	*Microbacterium hibisci*	+	+	-	-
W1_3/10	Iran, 1-29-11334	*Microbacterium hibisci*	+	+	-	-
W2_4/3	Turkey, Karakilcik	*Microbacterium paraoxydans*	+	+	+	-
C5_4/11	Brazil, BRS Guamirim	*Methylobacterium populi*	NT	+	-	-
CMAA1740	Brazil, Quartzo	*Paenarthrobacter ureafaciens*	+	+	+	+
C6_4/5	Brazil, Quartzo	*Paenarthrobacter ureafaciens*	-	+	-	NT
C6_2/6	Brazil, Quartzo	*Paenarthrobacter nicotivorans*	+	+	+	-
C5_3/11	Brazil, BRS Guamirim	*Paenibacillus esterisolvens*	+	+	+	-
W1_2/5	Iran, 1-29-11334	*Paenibacillus lautus*	NT	-	-	NT
W1_2/11	Iran, 1-29-11334	*Paenibacillus lautus*	NT	NT	-	-
W2_4/14	Turkey, Karakilcik	*Paenibacillus massiliensis*	NT	-	-	-
C6_3/10	Brazil, Quartzo	*Paenibacillus peoriae*	NT	+	-	+
CMAA1739	Turkey, Karakilcik	*Paenibacillus ottowii**	+	+	+	+
W1_2/12	Iran, 1-29-11334	*Pseudomonas taiwanensis*	+	+	-	NT
CMAA1741	Turkey, Karakilcik	*Pseudomonas inefficax**	+	+	+	+
C6_3/7	Brazil, Quartzo	*Rhodococcus erythropolis*	+	+	-	-
W2_4/10	Turkey, Karakilcik	*Streptomyces chartreusis*	+	+	-	-
W1_3/6	Iran, 1-29-11334	*Streptomyces cinereouber*	+	+	-	-
W1_3/5	Iran, 1-29-11334	*Streptomyces katrae*	+	+	-	-
W1_4/1	Iran, 1-29-11334	*Streptomyces virginiae*	+	NT	-	-
CMAA1738	Iran, 1-29-11334	*Streptomyces virginiae**	+	+	-	+
W2_4/12	Turkey, Karakilcik	*Streptomyces viridobrunneus*	+	+	-	-

(*) Indicates taxonomic assignment of the isolate from a fully sequenced bacterial genome. IAA = Indole-3-acetic acid; P = Phosphorus; N = Nitrogen. All results correspond to qualitative assays: "+" = positive; "−" = negative; NT = isolates not tested due to difficulties in colony growth.

Table S2. Pairwise comparisons of bacterial community assembly across treatments using the Pairwise Adonis test ($p < 0.05$).

Treatment	*p* value
Streptomyces virginiae CMAA1738	
B. sorokiniana vs. Control	0.121
B. sorokiniana vs. *Sv* + *B. sorokiniana*	0.002*
B. sorokiniana vs. Sv	0.003*
Control vs. *Sv* + *B. sorokiniana*	0.004*
Control vs. *Sv*	0.001*
Sv + *B. sorokiniana* vs. *Sv*	0.256
Paenibacillus ottowii CMAA1739	
B. sorokiniana vs. Control	0.124
B. sorokiniana vs. *Po* + *B. sorokiniana*	0.004*
B. sorokiniana vs. *Po*	0.003*
Control vs. *Po* + *B. sorokiana*	0.004*
Control vs. *Po*	0.002*
Po + *B. sorokiniana* vs. *Po*	0.244
Pseudomonas inefficax CMAA1741	
B. sorokiniana vs. Control	0.103
B. sorokiniana vs. *Pi* + *B. sorokiniana*	0.002*
B. sorokiniana vs. *Pi*	0.002*
Control vs. *Pi* + *B. sorokiniana*	0.009*
Control vs. *Pi*	0.005*
Pi + *B. sorokiniana* vs. *Pi*	0.010*

(*) Indicates significant difference ($p < 0.05$). Control = water treated plants; *Sv* = *Streptomyces virginiae* strain CMAA1738; *Po* = *Paenibacillus ottowii* strain CMAA1739; and *Pi* = *Pseudomonas inefficax* strain CMAA1741.

Table S3. Pairwise comparisons of fungal community assembly across treatments using the Pairwise Adonis test ($p < 0.05$).

**Treatment**	***p* value**
Streptomyces virginiae CMAA1738	
B. sorokiniana vs. Control	0.288
B. sorokiniana vs. *Sv* + *B. sorokiniana*	0.077
B. sorokiniana vs. *Sv*	0.010*
Control vs. *Sv* + *B. sorokiniana*	0.184
Control vs. *Sv*	0.087
Sv + *B. sorokiniana* vs. *Sv*	0.487
Paenibacillus ottowii CMAA1739	
B. sorokiniana vs. Control	0.284
B. sorokiniana vs. *Po* + *B. sorokiniana*	0.009*
B. sorokiniana vs. *Po*	0.002*
Control vs. *Po* + *B. sorokiana*	0.009*
Control vs. *Po*	0.008*
Po + *B. sorokiniana* vs. *Po*	0.026*
Pseudomonas inefficax CMAA1741	
B. sorokiniana vs. Control	0.271
B. sorokiniana vs. *Pi* + *B. sorokiniana*	0.064
B. sorokiniana vs. *Pi*	0.015*
Control vs. *Pi* + *B. sorokiniana*	0.182
Control vs. *Pi*	0.037*
Pi + *B. sorokiniana* vs. *Pi*	0.196

(*) Indicates significant difference ($p < 0.05$). Control = water treated plants; *Sv* = *Streptomyces virginiae* strain CMAA1738; *Po* = *Paenibacillus ottowii* strain CMAA1739; and *Pi* = *Pseudomonas inefficax* strain CMAA1741.
